# The ISWI Chromatin Remodeler Organizes the hsrω ncRNA–Containing Omega Speckle Nuclear Compartments

**DOI:** 10.1371/journal.pgen.1002096

**Published:** 2011-05-26

**Authors:** Maria C. Onorati, Sandra Lazzaro, Moushami Mallik, Antonia M. R. Ingrassia, Anna P. Carreca, Anand K. Singh, Deo Prakash Chaturvedi, Subhash C. Lakhotia, Davide F. V. Corona

**Affiliations:** 1 Dulbecco Telethon Institute, Università degli Studi di Palermo, Dipartimento STEMBIO – Sezione Biologia Cellulare, Palermo, Italy; 2 Cytogenetics Laboratory, Department of Zoology, Banaras Hindu University, Varanasi, India; Max-Planck-Institute of Immunobiology, Germany

## Abstract

The complexity in composition and function of the eukaryotic nucleus is achieved through its organization in specialized nuclear compartments. The *Drosophila* chromatin remodeling ATPase ISWI plays evolutionarily conserved roles in chromatin organization. Interestingly, *ISWI* genetically interacts with the *hsrω* gene, encoding multiple non-coding RNAs (ncRNA) essential, among other functions, for the assembly and organization of the omega speckles. The nucleoplasmic omega speckles play important functions in RNA metabolism, in normal and stressed cells, by regulating availability of hnRNPs and some other RNA processing proteins. Chromatin remodelers, as well as nuclear speckles and their associated ncRNAs, are emerging as important components of gene regulatory networks, although their functional connections have remained poorly defined. Here we provide multiple lines of evidence showing that the hsrω ncRNA interacts *in vivo* and *in vitro* with ISWI, regulating its ATPase activity. Remarkably, we found that the organization of nucleoplasmic omega speckles depends on ISWI function. Our findings highlight a novel role for chromatin remodelers in organization of nucleoplasmic compartments, providing the first example of interaction between an ATP-dependent chromatin remodeler and a large ncRNA.

## Author Summary

Chromatin structure and function are regulated by the concerted activity of covalent modifiers of chromatin, nucleosome remodeling factors, and several emerging classes of non-coding RNAs. ISWI is an evolutionarily conserved ATP-dependent chromatin remodeler playing essential roles in chromosome condensation, gene expression, and DNA replication. ISWI activity has been involved in a variety of nuclear functions including telomere silencing, stem cell renewal, neural morphogenesis, and epigenetic reprogramming. Using an *in vivo* assay to identify factors regulating ISWI activity in the model system *Drosophila melanogaster*, we recovered a genetic interaction between *ISWI* and *hsrω*. The *hsrω* gene encodes multiple non-coding RNAs (ncRNAs), of which the >10 kb nuclear hsrω-n RNA, with functional homolog in mammals, is essential for the assembly and organization of hnRNP-containing nucleoplasmic omega speckles. These special nuclear compartments play essential roles in the storage/sequestration of hnRNP family and other proteins, thus playing important roles in mRNA maturation and other regulatory processes. Here we show that the hsrω-n ncRNA interacts *in vivo* and *in vitro* with ISWI to directly regulate its ATPase activity. We also provide *in vivo* data showing that omega speckle nuclear organization depends on ISWI function, highlighting a novel role for chromatin remodelers in nuclear speckles organization.

## Introduction

ISWI, the catalytic subunit of several ATP-dependent chromatin remodeling complexes, is highly conserved during evolution and is essential for cell viability [1]. ISWI-containing complexes play central roles in DNA replication, gene expression and chromosome organization [2]. ISWI uses ATP hydrolysis to catalyze nucleosome spacing and sliding reactions [1]. Loss of *ISWI* function in *Drosophila* causes global transcription defects and dramatic alterations in higher-order chromatin structure, including decondensation of chromosomes [3], [4]. *In vitro* and *in vivo* studies in several model organisms have also shown the involvement of ISWI-containing complexes in other nuclear functions like telomere silencing, stem cell renewal, neural morphogenesis and epigenetic reprogramming during nuclear transfer in animal cloning [2], [5], [6]. The diverse functions associated with ISWI depend upon the ability of other cellular and nuclear factors to regulate its ATP-dependent chromatin remodeling activity [7]–[9]. In order to identify new regulators of ISWI function, we developed *in vivo* assays to identify genetic interactors of *ISWI* in *D.melanogaster* [10], [11]. Using an eye-based assay to identify factors antagonizing ISWI activity, we recovered, among other genes, a genetic interaction between *ISWI* and *hsrω* [10]. The *hsrω* gene is developmentally expressed in almost all cells types and is one of the most strongly induced heat shock genes in flies [12]–[14]. The *hsrω* locus encodes multiple non-coding RNAs (ncRNA), of which the large >10 kb nuclear species (hsrω-n) is essential for the assembly and organization of the hnRNP-containing omega speckles [14]. These specialized nuclear compartments are distinct from other nuclear speckles and are localized in the nucleoplasm close to chromatin edges [14]. Omega speckles play essential roles in storage and sequestration of members of the hnRNP family and other proteins involved in RNA processing and maturation in normal as well as environmentally or genotoxically stressed cells (for a list of hsrω interactors see [13]–[15]. Here we show that the hsrω ncRNA interacts *in vivo* and *in vitro* with ISWI to directly regulate its ATPase activity. Additionally, we provide *in vivo* data showing that omega speckle nuclear organization depends on ISWI function. Our work thus suggests that ISWI and the omega speckle associated hsrω ncRNAs interact and reciprocally affect each other's activities. Our findings highlight a novel role for chromatin remodelers in organization of nuclear speckles.

## Results

### *ISWI* Genetically Interacts with *hsrω*

**Figure 1:**
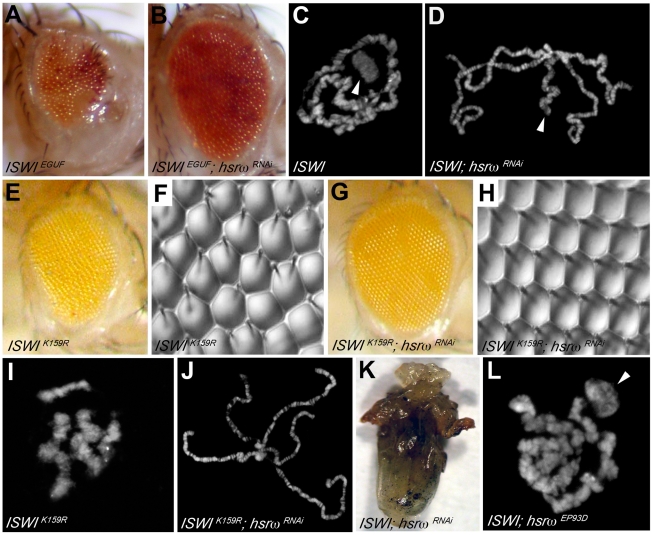
Loss of the hsrω ncRNA suppresses *ISWI* mutant defects. (A) The EGUF technique allows generation of adult eyes homozygous for a specific mutation in an otherwise heterozygous adult fly [34]. The *ISWI^EGUF^* eye is composed exclusively of clones that have lost *ISWI* activity, and is characterized by roughness and reduced size caused by loss of ommatidial boundaries or their orientation and reduced number of photoreceptors [11]. (B) Eye specific loss of *hsrω* function by RNAi (*ey-GAL4* driven *hsrω-RNAi* ^3^) [15] can suppress *ISWI^EGUF^* eye phenotypes with 100% penetrance. (C) Salivary gland polytene chromosomes from *ISWI^1^/ISWI^2^* trans-heterozygous *ISWI* null male larvae [4] (*ISWI*) are characterized by condensation defects, particularly of the X chromosome (arrowhead). (D) *ey-GAL4* driven *hsrω-RNAi^3^* suppresses the *ISWI* mutant male X chromosome condensation defects (arrowhead) with a 100% penetrance. In addition to the eye imaginal discs, the *eyeless* promoter is known to efficiently drive the expression of GAL4 also in salivary glands also [15]. (E) *ey-GAL4* driven mis-expression of the catalytically inactive *ISWI^K159R^* transgene produces reduced and rough eyes [4], [11]. (F) Nail polish imprint of eye mis-expressing *ISWI^K159R^* highlights ommatidial roughness. (G) Knock down of hsrω-n RNA by RNAi suppresses the *ISWI^K159R^* eye defects with a 100% penetrance. (H) Nail polish imprint of eye co-expressing *ISWI^K159R^* and *hsrω -RNAi^3^* transgenes confirms suppression of *ISWI^K159R^* ommatidial roughness. (I) *ey-GAL4* driven expression of *ISWI^K159R^* in salivary glands causes global decondensation of polytene chromosomes [3]. (J) hsrω-RNAi completely suppresses polytene chromosome condensation defects caused by *ISWI^K159R^* mis-expression. (K) The larval lethality associated with *ISWI*-null condition is partially rescued by the simultaneous down regulation of hsrω-n transcripts, as seen in this ventral view of a pharate dissected from a *ISWI^1^/ISWI^2^*; *Act5C-GAL4/hsrω RNAi* ^3^ dead pupa (also see Table S1B). (L) *Act5c-GAL4* driven *EP93D* [15] mediated over-expression of hsrω transcripts exaggerates the chromosome condensation defects of *ISWI*-null mutants (arrowhead points to the putative X chromosome).

Loss of *hsrω* function by RNAi [15] results in a striking amelioration of morphological defects in eyes exclusively composed of *ISWI*-null mitotic clones (Figure 1A, 1B and Figure S1A–S1D, S1J; Table S1A). Mutations in the *sqd* gene, which encodes the Squid hnRNP, a component of omega speckles, also suppresses *ISWI* mutant eye defects (Figure S1F–S1I and S1K; Table S1A) [10]. Absence of ISWI in larval salivary gland cells causes a dramatic decondensation of the male X polytene chromosome [4]. Remarkably, *hsrω-RNAi* suppresses the *ISWI* null male X chromosome condensation defects as well (Figure 1C, 1D). Tissue-specific mis-expression of the catalytically inactive *ISWI^K159R^* transgene also produces eye phenotypes and global chromosome decondensation [3], [4], [11]. Silencing of hsrω-n activity not only suppresses *ISWI^K159R^* eye phenotypes (Figure 1E–1H) but also restores normal polytene chromosome condensation (Figure 1I, 1J). In agreement with the above observations, the larval lethality of *ISWI*-null mutants is also partially suppressed by *hsrω*-*RNAi* (Figure 1K; Table S1B), strongly indicating that reduction of hsrω-n transcripts improves ISWI activity. On the other hand, over-expression of hsrω through the *hsrω^EP93D^* allele [15] antagonizes ISWI activity, resulting in enhanced chromosome condensation defects and eye phenotypes in *ISWI*-null background (Figure 1L and Figure S1E; Table S1A).

The suppression of chromosome condensation and eye defects in *ISWI* nulls by *hsrω-RNAi* is not due to a reduction in the efficiency of the GAL4/UAS driving system used to produce the *ISWI*-null and *ISWI^K159R^* mutant phenotypes (Figure S2A, S2B). Furthermore, the effect of hsrω-RNAi is highly specific for the loss of ISWI function (Figure S2C, S2D). Given the role played by omega speckles in nuclear RNA processing [13], we also checked if the levels of ISWI or ISWI*^K159R^* proteins and their corresponding mRNA were affected by hsrω-RNAi, which could account for the suppression of *ISWI*-null or *ISWI^K159R^* defects. However, depletion of hsrω transcripts by RNAi does not detectably affect ISWI protein or mRNA levels in either of these cases (Figure S3).

### Loss of ISWI Causes Global Defects in Omega Speckle Organization

**Figure 2:**
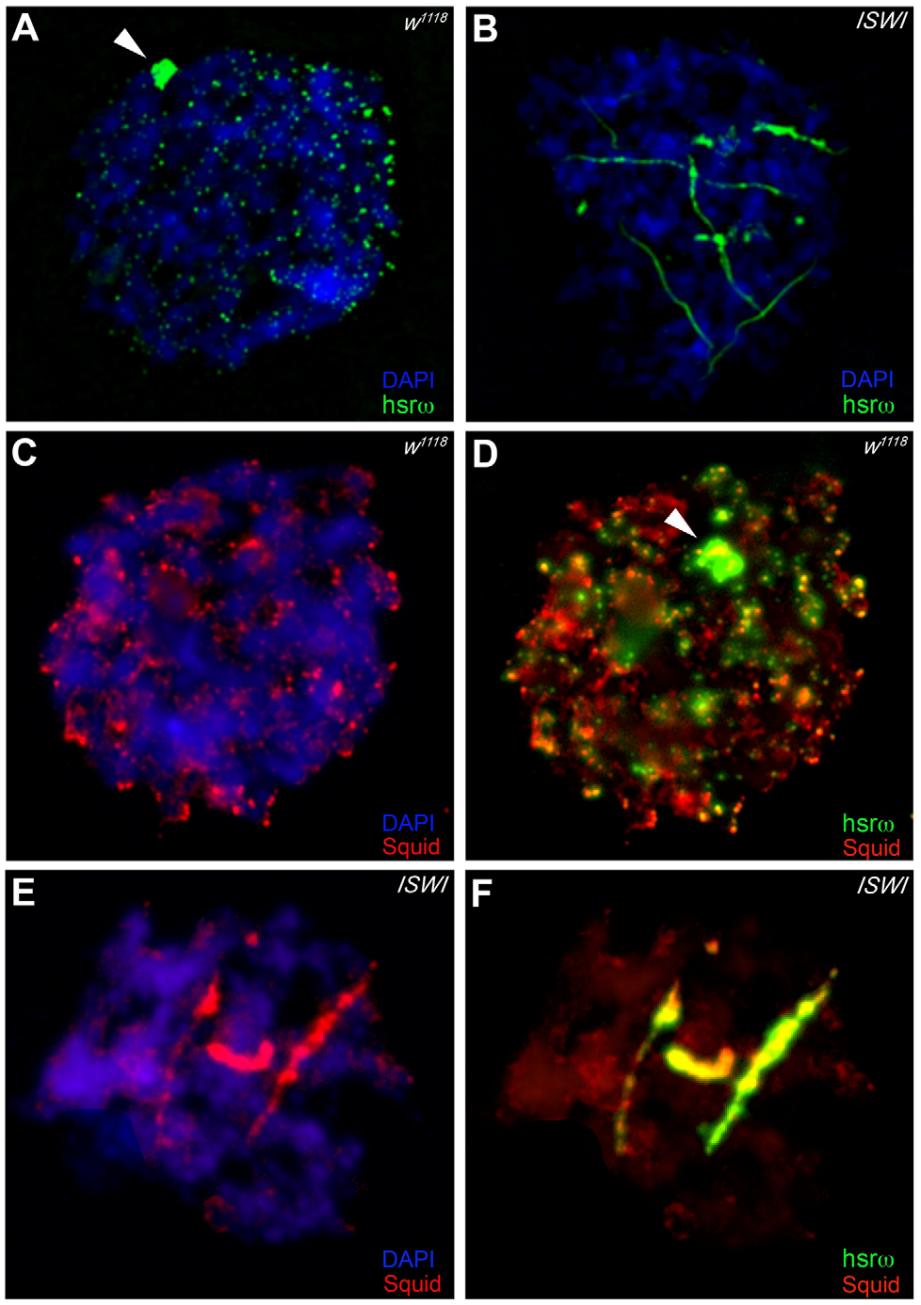
Loss of *ISWI* causes global defects in organization of omega speckles. (A) Confocal projection image of wild type (*w^1118^*) Malpighian tubule whole nucleus following fluorescent RNA in situ hybridization (FRISH) using the 280b tandem repeat riboprobe specific for the nuclear hsrω–n ncRNA [32]. The hsrω-n RNA localizes in omega speckles in the nucleoplasm mainly in the nuclear space not occupied by chromosomes, and at the 93D cytogenetic region corresponding to its site of transcription (arrowhead) [14]. (B) FRISH on *ISWI^1^/ISWI^2^* mutant (*ISWI*) larval Malpighian tubule nucleus reveals a dramatic change in the classic punctate pattern of omega speckles with a penentrance of 100%. The omega speckles in *ISWI* mutant cells are totally disorganized and form “trail”-like structures. DAPI stained DNA is shown in blue and hsrω–n RNA in green. (C) and (D) Besides its chromatin localization, the Squid protein largely colocalizes with the hsrω-n ncRNA [14] in omega speckles as seen after immunostaining for the Squid protein (red) and FRISH for hsrω-n RNA (green) on *w^1118^* Malpighian tubule whole nucleus. The 93D cytologenetic location is indicated by arrowhead. DNA was counterstained with DAPI (blue). (E) and (F) Immuno-FRISH of Squid (red) and hsrω-n RNA (green) on *ISWI^1^/ISWI^2^* mutant (*ISWI*) Malpighian tubule nucleus shows that besides the chromatin associated Squid, its nucleoplasmic fraction localizes in “trail”-like structures in *ISWI* mutant nuclei, instead of in the characteristic nucleoplasmic speckles. The Squid “trails” completely overlap with the hsrω signal, strongly indicating that loss of *ISWI* causes profound alteration in the organization not only of the hsrω-n ncRNA component but also of the associated Squid hnRNP. DNA was counterstained with DAPI (blue).

**Figure 3:**
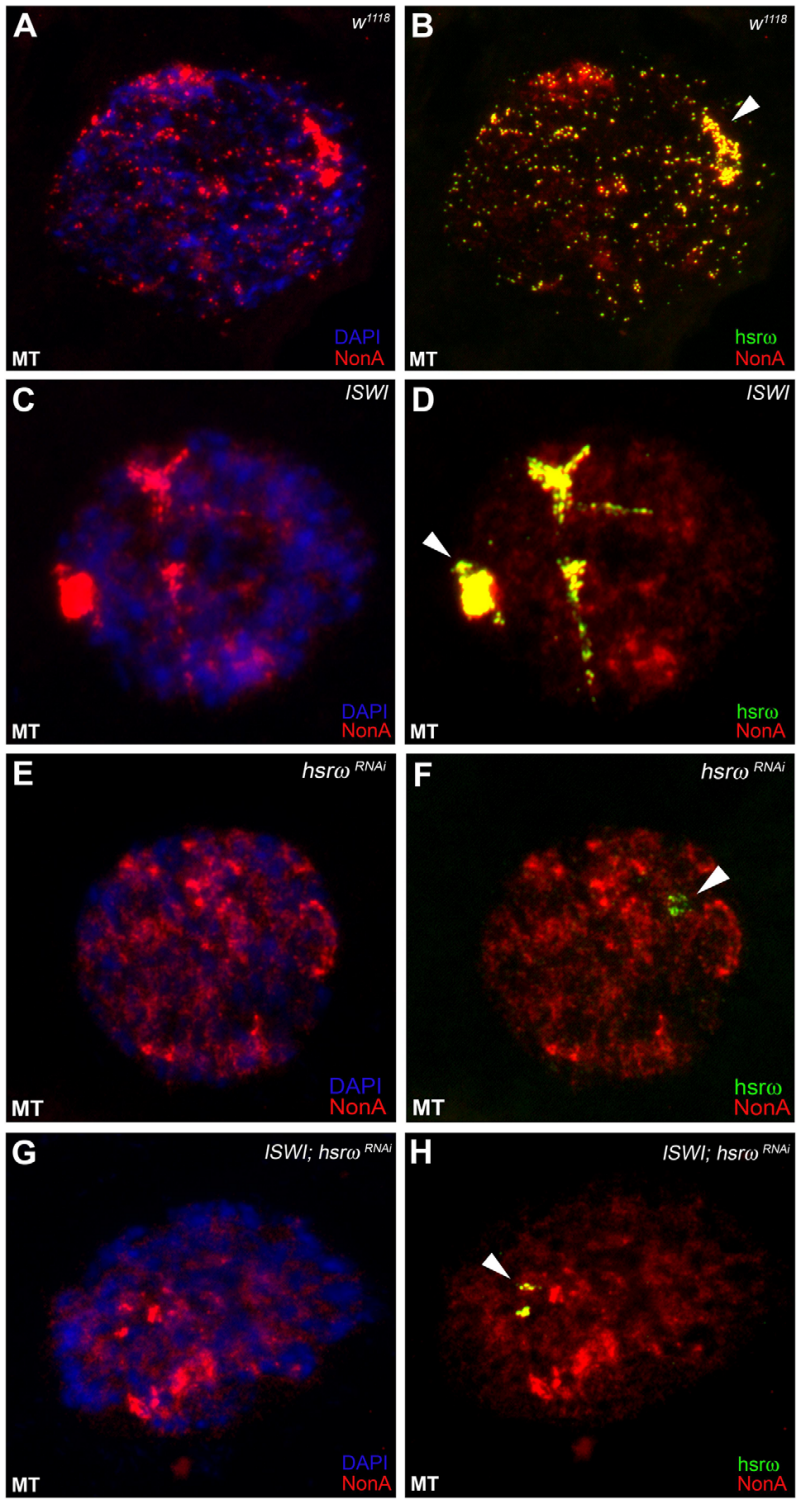
Defects in omega speckles in *ISWI* mutant cells are dependent on the presence of the hsrω ncRNA. (A) and (B) The NonA protein colocalizes with the hsrω-n ncRNA [14] in omega speckles as seen after immunostaining for the NonA protein (red) and FRISH for hsrω-n RNA (green) on *w^1118^* Malpighian tubule whole nucleus [14]. The 93D cytologenetic locus is indicated by the arrowhead. DNA was counterstained with DAPI (blue). (C) and (D) Immuno-FRISH of NonA (red) and hsrω-n RNA (green) on *ISWI^1^/ISWI^2^* mutant (*ISWI*) Malpighian tubule nucleus shows that the nucleoplasmic fraction of NonA also localizes in “trail”-like structures in *ISWI* mutant nuclei. The NonA “trails” completely overlap with the hsrω signal, strongly indicating that loss of *ISWI* causes profound alteration in the organization not only of the hsrω-n ncRNA component but also of omega speckle associated hnRNPs like Squid (see Figure 2C–2F) and Hrb87F (see Figure S4). (E) and (F) Down regulation of hsrω-n transcripts causes the omega speckle associated hnRNP components to diffuse in the nucleoplasm together with the absence of classic omega speckles. (G) and (H) *ISWI* null mutant condition does not result in formation of omega “trails” when the hsrω-n ncRNA is down regulated by RNAi.

In order to understand the molecular basis of the specific suppression of ISWI phenotypes by hsrω-RNAi, we examined the distribution and organization of omega speckles in the *ISWI* mutant third instar larval Malpighian tubule nuclei, which show abundant omega speckles using either RNA∶RNA *in situ* hybridization to hsrω-n ncRNA or immunostaining for some of the omega speckle associated hnRNPs [14]. Interestingly, the organization and distribution of omega speckles in *ISWI* mutants is profoundly altered when compared with wild type cells. Instead of typical speckles, the hsrω-n transcripts form “trail”-like structures in *ISWI*-null mutant nucleoplasm, indicating a severe defect in their maturation or organization (Figure 2A, 2B). Interestingly, Squid, NonA and other omega speckle associated hnRNPs also form “trail”-like structures in *ISWI* mutants (Figure 2C–2F, Figure 3A–3D, and Figure S4), which shows that distribution of not only the hsrω-n ncRNA but also of the omega speckle-associated hnRNPs is compromised in *ISWI* mutant nuclei. As shown earlier [15], the omega speckles do not form in the absence of hsrω-n transcripts and the omega speckle-associated hnRNPs remain diffused in the nucleoplasm (Figure 3E–3F). Interestingly, when the *ISWI* as well as hsrω-n ncRNA are absent, omega “trails” are not formed (Figure 3G–3H), strongly indicating that *ISWI* mutant specific omega “trails” are dependent on the presence of the hsrω-n ncRNA.

Analysis of live cells expressing a *Squid^GFP^* transgene [16] clearly identifies the GFP-positive “trails” in live *ISWI* mutant cells similar to those observed in fixed cells (Figure S5). This shows that the *ISWI* omega “trails” are not a fixation artifact. Significantly, comparable hsrω RNA “trails” were not seen (Figure S6) in the presence of other mutants like *jil1*, *ada2* and *gcn5* which also display chromosome condensation defects similar to those observed in the *ISWI* mutants [17], [18]. This excludes the possibility that the omega “trails” in *ISWI* mutant nuclei result from a “squeezing” effect of the nucleoplasm due to a massive “fallout” of chromatin associated proteins following global chromosome decondensation.

Studies in several model organisms have shown that ISWI plays a global role in transcriptional activation as well as repression [1], [3], [4]. Therefore, we examined if *ISWI* mutation altered the levels of hsrω-n ncRNA or the omega speckle-associated proteins. However, no significant difference in their levels was found between *ISWI* mutant and wild type cells (Figure S7). The >10 Kb hsrω-n ncRNA that organizes the omega speckles contains a small 0.7 Kb intron [14], [19]. It has been recently observed [20] that a spliced form of the hsrω-n transcript is also associated with the omega speckles. Therefore, we checked if the *ISWI* mutant condition affects splicing of this RNA which may result in the “trail”-like organization. RT-PCR and Northern blot analyses clearly indicate that *ISWI* mutation does not affect splicing of the hsrω-n ncRNA (Figure S8A–S8C).

In light of the significant role played by ISWI in gene expression, we checked whether an engulfment of the nuclear RNA export machinery in *ISWI* mutants affected RNA transport from nucleus, which in turn could modify the omega speckles into “trails”. *In situ* hybridization to cellular RNA with poly-dT probe did not reveal any difference in the nuclear vs cytoplasmic distribution of poly-A RNAs between wild type and *ISWI* mutant cells (Figure S8D). Thus, *ISWI* mutant nuclei do not seem to have a general RNA export defect, which could have been responsible for the observed omega “trails”.

Omega speckles are thought to provide a dynamic system to sequester and release specific RNA processing factors in normal as well as stressed cells [13]. Following heat shock, *hsrω* is one of the most highly transcribed genes [13], [21] and omega speckles coalesce into bigger growing clusters that finally get restricted to the *hsrω* gene locus, providing a dynamic sink for proteins that need to be transiently withdrawn from active nuclear compartments under stress conditions [14]. As already noted above, *ISWI* mutant condition causes the omega speckles to form nucleoplasmic “trails” in unstressed cells (Figure S9A, S9B). Although heat shock caused clustering of the omega speckles or “trails” in wild type and *ISWI* mutant cells, respectively, the numbers of clusters in the latter cells were much less (Figure S9C, S9D), suggesting that speckle dynamics under heat shock is also compromised because of *ISWI* mutant background. Finally, the “trail”-like organization of hsrω ncRNA and its associated proteins in *ISWI* mutants is not limited to Malpighian tubule or salivary gland polytene cells (Figure 2A, 2B and Figure S10A, S10B), but is also observed in *ISWI* mutant diploid cells (), indicating that disorganization of omega speckles is a general consequence of loss of *ISWI* function.

### Chromatin-Associated ISWI Interacts *In Vivo* and *In Vitro* with Nucleoplasm-Associated Omega Speckles

**Figure 4:**
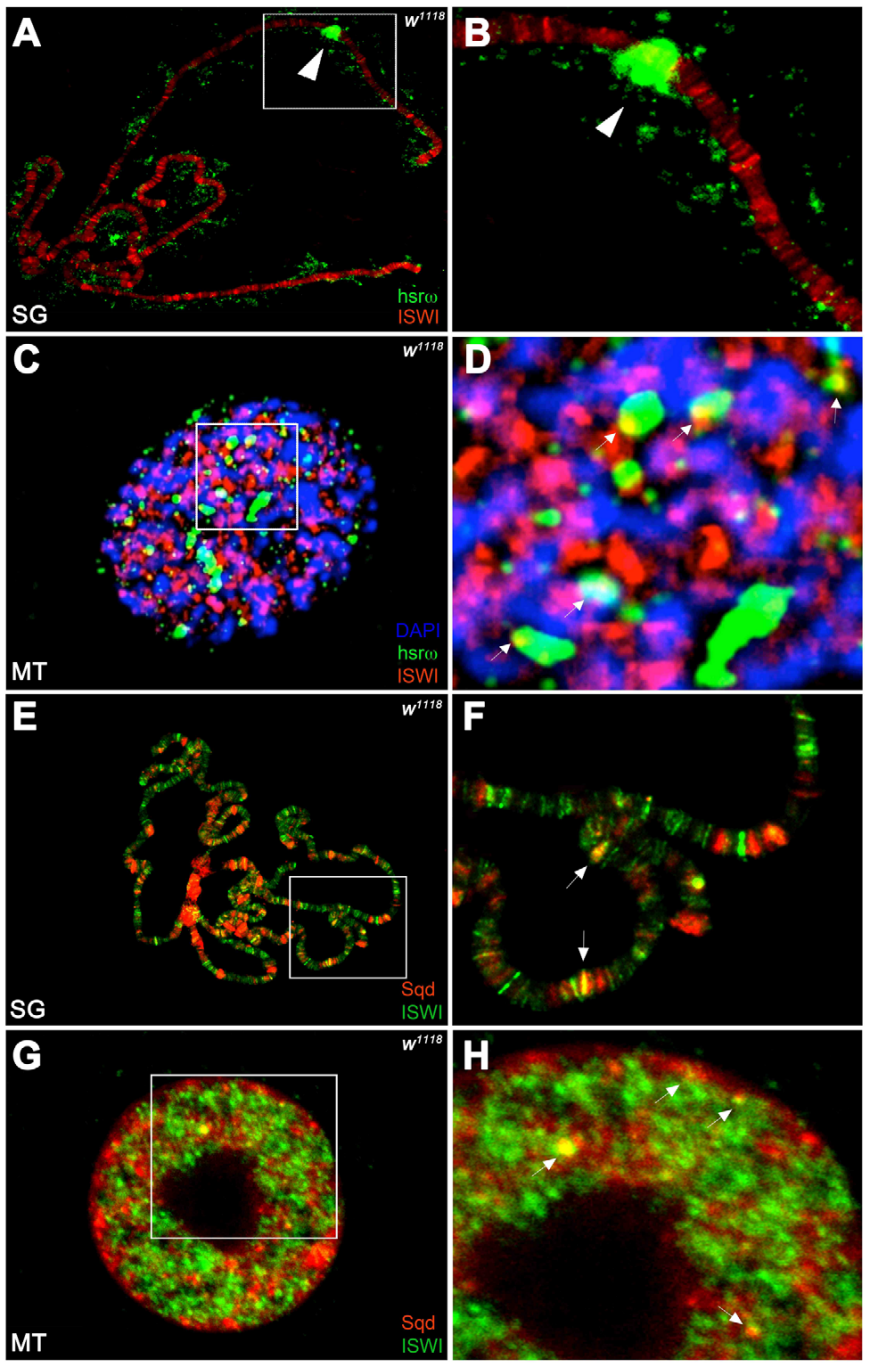
ISWI interacts *in vivo* with omega speckles. (A) Immunostaining for the ISWI protein (red) combined with FRISH for hsrω-n RNA (green) on wild type (*w^1118^*) salivary gland (SG) squashed nuclei shows omega speckles decorating polytene chromosome edges without any significant overlap with chromosome-bound ISWI. Arrowhead indicates the 93D cytologenetic location. (B) A magnified image of ISWI and hsrω-n signals corresponding to the white boxed area in A. (C) Immuno-FRISH confocal section of wild type (*w^1118^*) Malpighian tubule (MT) whole nucleus shows that ISWI (red) and hsrω-n (green) partially overlap (yellow areas) at several sites. DAPI stained DNA is in blue. (D) A magnified view of DAPI (blue), ISWI (red) and hsrω-n RNA (green) signals corresponding to the white boxed area in C shows typical examples of partial overlap (yellow, white arrowheads) between ISWI and hsrω-n RNA. (E) Double immunostaining for the ISWI (green) and Squid (red) proteins on *w^1118^* salivary gland (SG) squashed nucleus, shows that with the exception of a few sites, there is little overlap between the two proteins on polytene chromosomes. DAPI stained DNA is in blue. (F) A magnified image of Squid and ISWI signals corresponding to the white boxed area in E shows the few co-immunostained (yellow) regions (white arrows). (G) Double immunostained confocal section to show partial overlap between ISWI (green) and Squid (red) proteins in the nucleoplasm in *w^1118^* Malpighian tubule (MT) whole nucleus. The black empty area corresponds to the nucleolus. (H) A magnified image of Squid and ISWI signals corresponding to the white boxed area in G shows representative sites where ISWI and Squid proteins partially overlap (white arrows).

Unlike the association of ISWI with different bands and interbands on polytene chromosomes [4], [11], the hsrω-n ncRNA localizes in the nucleoplasm in proximity or at the edges of chromosome spreads, without any apparent overlap with the chromatin associated ISWI (Figure 4A, 4B). However, examination of confocal images of intact nuclei revealed some chromosome-nucleoplasm sites where ISWI and the hsrω-n ncRNA are adjacent and seem to form connecting bridges between nucleoplasm and chromatin (Figure 4C, 4D). Barring a few exceptions, Squid and other omega speckles associated hnRNPs also showed no overlap with ISWI on polytene chromosome spreads (Figure 4E, 4F and Figure S11A, S11B). Significantly, like the hsrω ncRNA they too were found to partially overlap with ISWI in several nucleoplasmic foci in intact nuclei (see Figure 4G, 4H and Figure S11C, S11D), suggesting that ISWI may indeed partially interact directly or indirectly, at least transiently, with omega speckles in the three-dimensional nuclear space.

**Figure 5:**
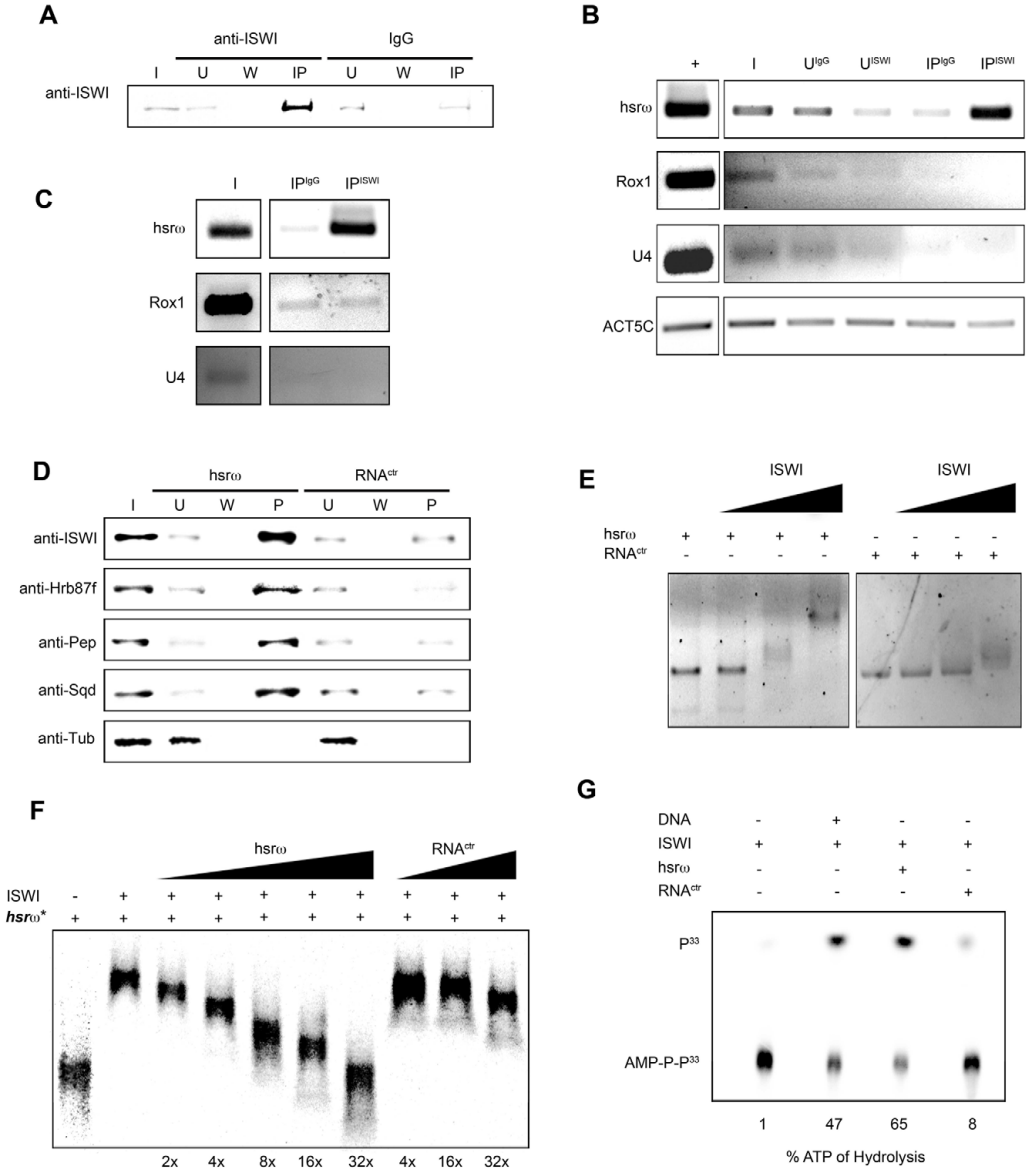
ISWI physically and functionally interacts with the hsrω-n ncRNA. (A) RNA-Immunoprecipitation (RIP) was conducted on larval nuclear extracts [35] using an affinity purified ISWI antibody [4]. The immunoprecipitated material was split in two parts. The first aliquot was analyzed by Western blotting using the ISWI antibody. Generic rabbit IgG was used as control. I = Input, U = Unbound, W = Wash, IP = Immunoprecipitated material. (B) Total RNA was extracted from the second immunoprecipitated aliquot for RT-PCR using primers that specifically amplify the 280b tandem repeat characteristic of the nuclear hsrω–n ncRNA. RT-PCR amplification of the U4 and Rox1 ncRNAs was also carried out using specific primers for each to assess the specificity of amplification of hsrω–n repeat unit in the ISWI immunoprecipitate. RT-PCR amplicon generated with primers for Act5C RNA was used to normalize the RT-PCR signals. + = RT-PCR positive control amplification, I = Input, U^ISWI^ = Unbound from anti-ISWI RIP, U^IgG^ = Unbound from IgG RIP, IP^ISWI^ = Immunoprecipitated material from anti-ISWI RIP, IP^IgG^ = Immunoprecipitated material from IgG RIP. (C) To validate the physical interaction between ISWI and hsrω observed by RIP on native larval nuclear extracts (see B), CLIP (Cross-Linking & Immuno Precipitation) was carried out with affinity purified anti-ISWI antibody [4] on fixed larval nuclear extracts. I = Input, IP^ISWI^ = Immunoprecipitated material from anti-ISWI CLIP, IP^IgG^ = Immunoprecipitated material from IgG CLIP. The immunoprecipitate was analyzed by RT-PCR using primers for the 280b tandem repeat unit of the nuclear hsrω–n ncRNA; primers amplifying the U4 and Rox1 ncRNAs were used as specificity controls. (D) Immobilized hsrω-n ncRNA and a generic control RNA were used as baits to pull down protein complexes from native larval nuclear extracts. Pulled down material was detected by Western blotting using antibodies against ISWI, Hrb87F, Pep and Sqd; Tubulin (Tub) was used as the loading control. (E) Gel mobility assay of the *in vitro* transcribed 280b tandem repeat unit of the hsrω-n ncRNA was carried out in the presence of increasing amounts of recombinant full length ISWI, with the ISWI/RNA molar ratios 0∶1, 1∶1, 5∶1 and 10∶1 nmoles, respectively. A generic RNA (RNA^ctr^) of approximately 300 bp was used as control. (F) Gel mobility assay was carried out with P^33^ radiolabeled *in vitro* transcribed hsrω-n ncRNA (hsrω*: 280b tandem repeat unit) in the presence of a fixed amount of recombinant full length ISWI (molar ratio ISWI/hsrω* of 8∶1). Increasing amounts of cold hsrω 280b ncRNA (2×, 4×, 8×, 16×, 32× fold excess with respect to hsrω*) specifically compete the ISWI binding with radiolabeld hsrω RNA by changing its mobility. The same fold excess of the control generic RNA (RNA^ctr^) very poorly changes the mobility of the retarded ISWI/hsrω* complex. It is important to note that when an excess of cold hrsω ncRNA is added in the ISWI/hsrω* binding reaction, the retarded complex changes its mobility instead of going away to form free probe at the bottom of the gel. Our data suggest that more than one ISWI protein binds one hrsω ncRNA molecule, so that as the amount of cold hsrω ncRNA increases, fewer ISWI molecules bind with each unit and thereby retard its movement to a lesser extent. (G) Stimulation of the ATPase activity of ISWI by generic DNA, *in vitro* transcribed 280b repeat unit of the hsrω-n or a generic RNA (RNA^ctr^) was assayed *in vitro* by thin layer chromatography in the presence of radioactive ATP-γ^33P^. Stimulation of the ATPase activity by the distinct nucleic acids assayed is noted below each lane as percentage of the ATP hydrolysed (P^33^ = P^33^ radioactive labeled hydrolyzed gamma phosphate, AMP-P-P^33^ = radioactive labeled non-hydrolyzed ATP).

In order to directly investigate whether the chromatin remodeling factor ISWI physically interacts with omega speckles, we used an affinity purified ISWI antibody [4] to conduct classic RNA immunoprecipitation. Our semi-quantitative RT-PCR analysis revealed the presence of hsrω-n ncRNA in larval nuclear extracts immunoprecipitated with ISWI antibody (Figure 5A, 5B). To rule out a non-specific association of ncRNAs with ISWI, we used the same immunoprecipitate to detect U4 and Rox1 [22] ncRNAs by RT-PCR. Significantly, neither of these two otherwise abundant ncRNAs were detectable (Figure 5A, 5B) in the mmunoprecipitate. This confirms the specificity of the physical interaction between ISWI and hsrω-n RNA in native larval extracts. Further, to exclude the possibility that the physical association observed between ISWI and hsrω was due to fortuitous interactions occurring during nuclear extract preparation, we conducted the CLIP assay (Cross-Linking & Immuno Precipitation) using the affinity purified anti-ISWI antibody [4] on fixed larval nuclear extracts. The CLIP data confirmed a highly specific interaction between ISWI and the hsrω ncRNA in the nucleus (Figure 5C), as observed with the native extracts (Figure 5B). Moreover, as shown in Figure 5D, RNA pull down assay confirmed that ISWI is also specifically pulled down by immobilized hsrω-n ncRNA along with the other known omega speckles associated hnRNPs [13] while a control generic RNA does not pull down ISWI or the other hnRNPs (Figure 5D).

Classic gel shift assay using *in vitro* transcribed hsrω-n ncRNA repeat unit (280b) and full length recombinant ISWI clearly shows that ISWI effectively retards hsrω-n ncRNA mobility, but that of a generic control RNA is retarded poorly (Figure 5E). Moreover, the mobility shift of the hsrω-n RNA by ISWI binding is specifically competed by hsrω-n but not by a generic RNA (Figure 5F). This further confirms the specific nature of ISWI/hsrω physical interaction *in vitro*.

A functional significance of the physical interaction between ISWI and hsrω-n ncRNA is indicated by the stimulation of ISWI ATPase activity. Remarkably, as also reported previously [23], while the generic control RNA very poorly stimulates the ISWI ATPase activity, the hsrω-n ncRNA was found to specifically stimulate the ISWI ATPase activity to levels greater than those normally seen with DNA but lower than nucleosome-stimulation (Figure 5G) [23].

The 280b hsrω-n nuclear ncRNA repeat unit used for the binding and ATPase assays is predicted to organize into a stable double stranded RNA molecule containing a few loops (Figure S12). This secondary organization is common to many RNAs, but this structure is also reminiscent of a double stranded DNA molecule. Therefore, it remained possible that the recognition of a double stranded nucleic acid (RNA or DNA) may provide a basis for the observed binding and stimulation of ATPase activity of ISWI by the hsrω-n ncRNA. When we checked the ability of the double stranded DNA sequence encoding the hsrωncRNA to elicit ISWI ATPase activity, we found that ISWI was stimulated to levels similar to those reported for other generic linear double stranded DNA molecules [23] (Figure S13A). Furthermore, co-presence of hsrω-n ncRNA and nucleosomes in a classic ATPase assay with ISWI clearly shows that both substrates compete for ISWI binding and its ATPase activity stimulation (Figure S13B).

**Figure 6:**
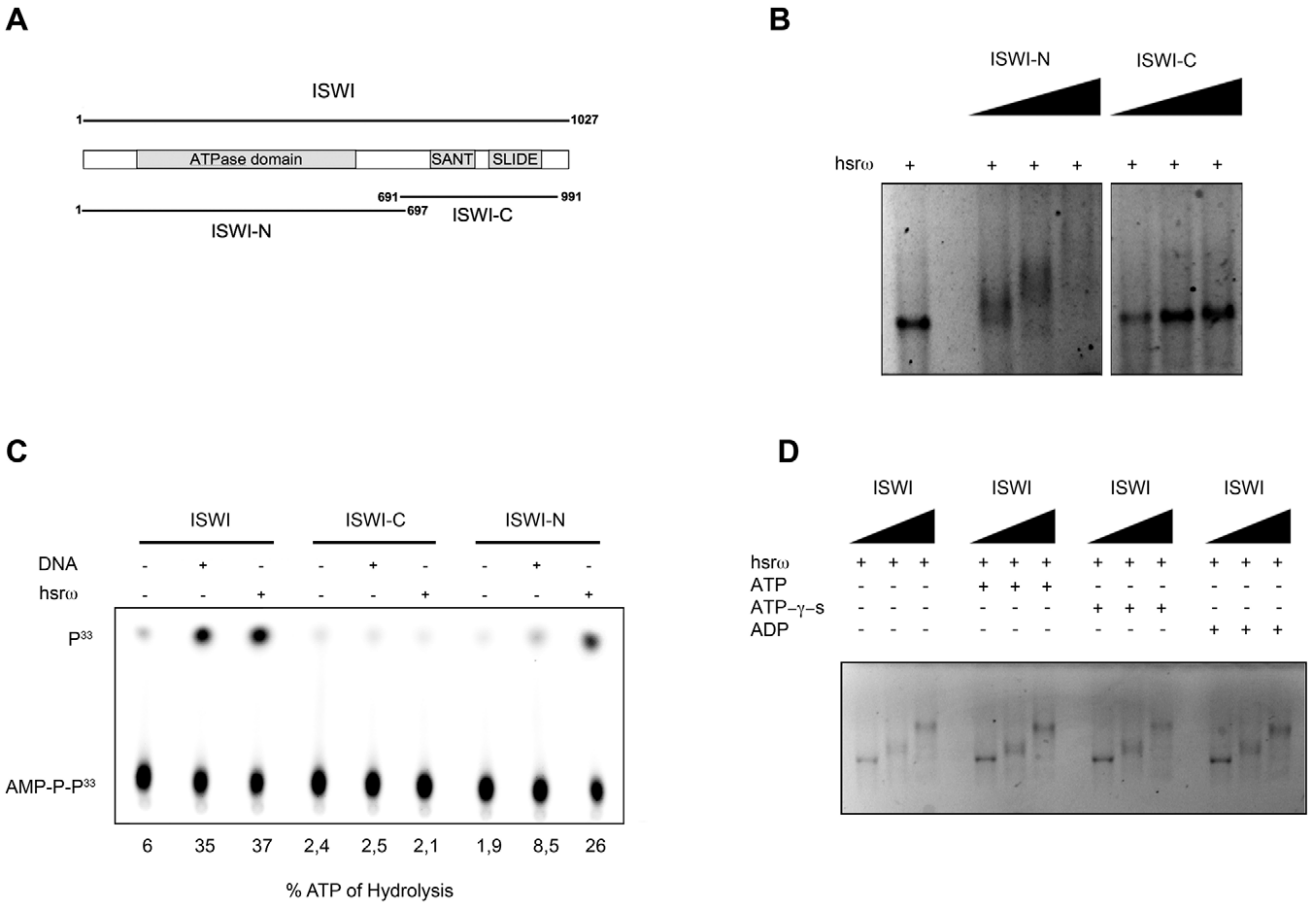
The N-terminal portion of ISWI interacts with the hsrω-n ncRNA, but its ATPase activity is not necessary for binding. (A) Schematic representation of the two known functional domains of ISWI, the amino-terminal (ISWI-N) comprising the ATPase domain and the carboxy-terminal (ISWI-C) that specifically recognizes DNA in the context of nucleosomes through the SANT and SLIDE domains [24]. (B) Gel mobility shift assay of the 280b hsrω-n repeat unit transcript in the presence of increasing amount of ISWI-N or ISWI-C with ISWI/RNA molar ratios of 0∶1, 5∶1, 10∶1 and 20∶1 nmoles, respectively. (C) The ATPase activity of full length ISWI or its sub-domains (ISWI-N or ISWI-C) in the presence of DNA or the 280b hsrω-n repeat unit transcript was assayed *in vitro* by thin layer chromatography. Percentage of ATP hydrolysis, calculated as before (Figure 5G) is noted below each lane. (D) Gel mobility shift assay with hsrω-n ncRNA, in the presence of ATP, ATP-γ-S or ADP, with increasing amount of recombinant full length ISWI (ISWI/RNA molar ratios 0∶1, 5∶1 and 10∶1 nmoles, respectively).

The ISWI protein has two functional domains (Figure 6A), the N-terminal (ISWI-N) ATPase domain and the C-terminal (ISWI-C) nucleosomal DNA recognizing domain [24]. Results presented in Figure 6B and 6C, show that the hsrω-n binds with the ISWI-N fragment and stimulate its ATPase activity, suggesting that ISWI could interact with hsrω-n ncRNA through its ATPase domain. Therefore, we further checked if the presence of ATP, ATP-γ-S (a non-hydrolizable form of ATP) or ADP could affect ISWI binding or determine a conformational change in the ISWI/hsrω complexes resolved by gel shift. Our data show that all the three nucleotides have no effect on ISWI binding (Figure 6D), probably suggesting that the ATPase activity of ISWI may not be necessary for physical interaction between ISWI and hsrω RNA.

## Discussion

Factors that coordinate nuclear activities occurring on chromatin and the nucleoplasmic compartments remain unidentified and uncharacterized. Therefore, an important open question in nuclear organization field is how nuclear speckles localize and organize themselves near transcriptionally active genes to cross talk with chromatin factors for processing of the nascent RNAs. Our data indicate that ISWI may provide a functional ‘bridge’ between chromatin and nuclear speckle compartments. Indeed, ISWI can directly or indirectly contact the omega speckles in intact nuclei, through hsrω-n ncRNA or some of the associated hnRNPs. Our confocal analysis suggested a functional ‘bridge’ between a chromatin factor (ISWI) and nucleoplasmic omega speckle components (hsrω ncRNA and hnRNPs). However, not all omega speckles show partial overlap with ISWI. Indeed, these molecular “bridges” between chromatin and nucleoplasm are probably transient, since time-lapse movies on live cells with fluorescently tagged chromatin and omega-speckle components clearly show very high mobility of these speckles (see Video S1), which probably may explain the absence of classic co-localization between ISWI and omega speckle components.

The observed direct physical interaction between ISWI and hsrω-n ncRNA together with the stimulation of ISWI-ATPase activity in light of the partial overlap revealed by confocal microscopy suggests that ISWI may interact with hsrω-forming speckles only transiently, probably to help the hsrω ncRNA to properly associate with or release the various omega speckle-associated hnRNPs. Loss of *ISWI* may impair the correct maturation, organization or localization of omega speckles resulting in the observed omega “trail” phenotype.

Our data also provide a possible explanation for the suppression of *ISWI* defects by hsrω-RNAi. In *ISWI* mutants carrying normal levels of hsrω transcripts, the limited maternally derived ISWI [3] is shared between chromatin remodelling and omega speckle organization reactions so that its sub-threshold levels in either compartments severely compromises both functions (see Video S2). However, when hsrω transcript levels are reduced by RNAi in *ISWI* null background, most of the maternal ISWI may become available for chromatin remodelling reactions, so that a minimal threshold level of chromosome organization can be achieved. This would permit initiation of close to normal developmental gene activity programs resulting in suppression of the ISWI eye and chromosome defects or in the postponement of the larval lethality to pupal stage. Additionally, it is known that when *hsrω* ncRNA is down regulated through RNAi, levels of free hnRNPs and other chromatin factors (i.e. CBP) are also elevated [25]. Therefore, we cannot formally exclude the possibility that these changes may also counteract *ISWI* defects by as yet unknown mechanisms.

Our work provides the first example of modulation of an ATP-dependent chromatin remodeler by a ncRNA, and to our knowledge the first *in vivo* and *in vitro* demonstration of a role of chromatin remodeler in organization of a nuclear compartment. However, the mechanism underlying stimulation of the ATPase activity of ISWI by the hsrω-n ncRNA, which may facilitate the organization of omega speckles, remains to be understood. Given the evolutionary derivation of the ISWI ATPase-domain from RNA-helicase-domains [1], a provocative hypothesis is that ISWI could “remodel” speckles by structurally helping the assembly or release of specific hnRNPs with the hsrω-n ncRNA to generate mature omega speckles. Chromatin remodelers, nuclear speckles and their associated long ncRNAs are emerging as essential components of gene regulatory networks, and their deregulation may underlie complex diseases [15], [25]–[27]. The functional homology of the human noncoding *sat III* transcripts with the *Drosophila* hsrω ncRNA [13], [27], highlights the relevance and translational significance of studies unraveling the functional connections between ncRNA-containing nuclear compartments and chromatin remodelers.

## Materials and Methods

### Fly Strains and Genetic Interaction

Flies were raised at 22°C on K12 medium [28]. Unless otherwise stated, strains were obtained from Bloomington, Szeged or VDRC (Vienna Drosophila RNAi Center). Genetic tests for dominant modifier (enhancement or suppression) of *ISWI-EGUF* and *ISWI^K159R^* phenotypes were conducted as previously described [10], [11]. The tissue specific expression of the *UAS-ISWI^K159R^* [4], the *UAS-hsrωRNAi^3^* and the *EP93D* transgenic lines [15] was obtained with *ey-GAL4* (for eyes and larval salivary glands) or *Act5C-GAL4* driver (for larval Malpighian tubules and testis). The surface architecture of adult eyes was examined by the nail polish imprint method [26]. For the larval lethality assay, numbers of larvae of different genotypes that pupated and the numbers of pupae emerging as flies in a given cross were separately counted.

### Antibodies, Plasmids, and RNA Probes

Mouse monoclonal antibodies against the following proteins were used at the indicated dilutions: Hrb87F (P11) [14] dilution 1∶5 for IF and 1∶100 for WB; Squid (1B11) [29] dilution 1∶100 for IF and 1∶2000 for WB; NonA [30] dilution 1∶50 for IF and 1∶1000 for WB; PEP [31] dilution 1∶2000 for WB. Affinity purified rabbit ISWI antibody [4] was diluted 1∶200 for IF and 1∶2000 for WB. FITC- and Rhodamine- conjugated anti-mouse and anti-rabbit secondary antibodies (Jackson Immuno Research) were diluted 1∶200 for IF and 1∶2000 for WB, respectively. The biotin-labeled anti-sense hsrω-n RNA 280b riboprobe was generated from the *pDRM30* plasmid [32] and used for FRISH. For gel mobility assays the sense hsrω-n RNA riboprobe was generated from the same plasmid.

### Immunofluorescence, FRISH, and ImmunoFRISH

Single and double immunofluorescence on polytene chromosome spreads were conducted as previously described [11]. Larval tissues (salivary glands, Malpighian tubules and testis) were dissected from third-instar larvae grown at 22°C. Fully or partially squashed tissue preparations were used for FRISH and Immuno-FRISH assays as previously described [14] with some modifications (Text S1).

### Protein Extraction and Western Blotting (WB)

Total proteins from salivary glands and Malpighian tubules were extracted as previously described [11]. The SDS-PAGE separated proteins were transferred onto nitrocellulose membrane (Whatman Schleicher & Schuell) for Western detection using SuperSignal West Femto substrate (Pierce). Chemiluminescent signals were acquired with the ChemiDoc XRS imager (BioRad).

### RNA–Immunoprecipitation

Native larval nuclear protein extracts from third instar *w^1118^* larvae were prepared as previously described [11] and RNA-immunoprecipitations were conducted as published earlier [33] with small modifications (Text S1).

### ISWI/hsrω-n Gel Mobility Assay

Recombinant full length ISWI or ISWI-N or ISWI-C proteins [23], [24] were incubated with *in vitro* transcribed sense 280b tandem repeat unit of the hsrω-n ncRNA or a generic RNA of the same size (RNA^ctr^, Roche) as a control, in increasing ratios of 1∶1, 5∶1,10∶1 and 20∶1 nmoles. The hsrω-n ncRNA or the RNA^ctr^ were incubated with the desired protein for 30 min at 25°C in RB2 buffer (20% Glycerol, 0.2 mM EDTA, 20 mM Tris-HCl pH 7.5, 1 mM MgCl_2_, 150 mM NaCl, 1 mM DTT and RNAsin). After incubation, the RNA/protein complexes were resolved on 1.4% agarose gel in 0.5× TBE at 4°C for 105 minutes at 70 volts. RNA molecules were visualized by ethidium bromide staining. ATP, ATP-γ-S and ADP (Roche) were added in the gel shift assay at a final concentration of 100 µM. Excess of cold hsrω-n repeat unit or a generic RNA^ctr^ transcript was used as competitor for ISWI/hsrω binding detected by gel mobility shift using P^33^ radiolabeled hsrω280b sense repeat unit and recombinant ISWI. RNA/protein complexes were resolved as above. After gel drying, RNA/protein complexes were visualized using the BioRad Phosphoimager system.

### ATPase Assay

ATPase assay was conducted as previously described [23]. Extent of ATP hydrolysis was calculated with the following formula [P^33^/(P^33^+AMP-P−P^33^)]*100 (Figure 5G). The ATPase activity of 4 nmoles of full length ISWI was assayed for 1 hour; 4 nmoles of ISWI-N and ISWI-C were assayed for 30 minutes in the presence of 2 nmole of either supercoiled plasmid DNA, 280 bp hsrω-repeat unit encoding double stranded DNA, hsrω-n 280 bp tandem repeat ncRNA or a 300 bp generic RNA (RNA^ctr^; Roche) as a control.

## Supporting Information

Figure S1 Genetic interactions between *ISWI*, *hsrω* and *sqd*. (A, B, C and D) Eye phenotypes resulting from eye homozygous for the *ISWI^2^* allele (*ISWI^EGUF^*) [11] are suppressed by the *hsrω* alleles, *hsrω^EP3115^* [10], *hsrω^e01850^*, *hsrω ^EP3037^* and *hsrω ^DG16301^*, respectively, thus reconfirming the genetic interaction between *ISWI* and *hsrω* as reported earlier [10] (also see Table S1A). (E) *ey-GAL4* directed over-expression of *hsrω* through the *hsrω^EP93D^* allele [15] enhances *ISWI^EGUF^* eye phenotype, suggesting that an excess of hsrω transcripts antagonizes ISWI function (also see Table S1A). (F, G, H and I) *ISWI^EGUF^* eye defects are suppressed by the *sqd^EP3631^*, *sqd^c04803^*, *sqd ^e01416^* and *sqd ^f01931^* alleles, thus revalidating a genetic interaction between *ISWI* and *sqd* [10] (also see Table S1A). (J and K) Schematic genetic map showing locations of the *hsrω* and *sqd* insertion alleles, respectively, used in the above *ISWI^EGUF^* assay. Introns are displayed as thin lines, exons by filled boxes. Noncoding regions are in grey and the coding parts are shown in orange. Alternatively spliced transcripts of the *sqd* gene, as reported on the FlyBase (www.flybase.org), are also shown.
(TIF)

Figure S2 The suppression of *ISWI* phenotype by *hsrω-RNAi* is highly specific. (A and B) Nuclear expression of GFP from a UAS-GFP^nls^ transgene is not reduced when the hsrω ncRNA is knocked down by RNAi, indicating that loss of *hsrω* function does not interfere with the GAL4/UAS driving system, as also previously shown [15], [26]. (C) The *brm* gene encodes a chromatin remodeler but with functions opposing ISWI [16]. Eye specific mis-expression of the catalytically inactive *brm^K804R^* allele produces rough and reduced eyes that are reminiscent of those obtained with the catalytically inactive *ISWI^K159R^* allele. (D) Unlike the suppression of *ISWI^K159R^* phenotype (see Figure 1E–1H), eye-specific expression of hsrω-RNAi does not suppress *brm^K804R^* eye defects, strongly indicating that the suppression of *ISWI^K159R^* defects by hsrω-RNAi is specific.
(TIF)

Figure S3 The hsrω-RNAi does not cause any change in ISWI protein as well as mRNA stability. (A) Western blots of salivary gland nuclear extracts [35] from wild type (*w^1118^*), *ISWI* null (*ISWI^1^/ISWI^2^*), and *ISWI*; *hsrω* double mutants (*ISWI^1^/ISWI^2^*; *ey-Gal4/UAS-hsrω-RNAi* ^3^) challenged with ISWI (anti-ISWI) [4] and Tubulin (anti-Tub; SIGMA) antibodies. The level of ISWI positive signal relative to *w^1118^* extract (as percentage) is noted below each lane. (B) Western blot of salivary gland nuclear extracts [35] derived from *w^1118^*, HA-tagged *ISWI^K159R^* mis-expressing mutants (*ey-Gal4/+; UAS-HA-ISWI* ^K1*59R*^ */+*), or form glands that co-express, HA-tagged *ISWI^K159R^* and *hsrω*-*RNAi* (*ey-Gal4/+; UAS-HA-ISWI* ^K159R^/*UAS-hsrωRNAi* ^3^) transgenes challenged with ISWI (anti-ISWI) [4], HA epitope (anti-HA; ROCHE) or Tubulin (anti-Tub; SIGMA) antibodies. Level of ISWI positive signal relative to *w^1118^* extract (as percentage) is noted below each lane. (C) RT-PCR analysis of total RNA extracted from salivary glands derived from *w^1118^*, *ISWI*-null (*ISWI^1^/ISWI^2^*), HA-tagged *ISWI^K159R^* expressing mutants alone (*ey-GAL4/+; UAS-HA-ISWI* ^K1*59R*^ */+*), *hsrω* knock down alone (*ey-GAL4/+; UAS-hsrωRNAi^3^/+*), or glands co-expressing HA-tagged *ISWI^K159R^* and *hsrω-RNAi* (*eyGal4/+; UAS-HA-ISWI* ^K159R^/*UAS-hsrωRNAi^3^*) and finally from glands that are *ISWI*; *hsrω* double mutants (*ISWI^1^/ISWI^2^*; *ey-GAL4/UAS-hsrωRNAi* ^3^) using primers specific for ISWI, hsrω-n or the Act5C transcripts. The quantification of PCR amplified ISWI mRNA signal relative to wild type (*w^1118^*) extract is shown in percentage below each lane.
(TIF)

Figure S4 Hrb87F forms omega trails in *ISWI* mutant nuclei. The Hrb87F protein also co-localizes in the nucleoplasm with the hsrω-n ncRNA in the nucleoplasmic omega speckles [14] as shown by immunostaining for Hrb87F (red) combined with FRISH for hsrω-n (green) ncRNA on intact wild type (w^1118^) Malpighian tubule nuclei. Immuno-FRISH of Hrb87F (red) and hsrω-n ncRNA (green) on intact *ISWI^1^/ISWI^2^* (*ISWI*) Malpighian tubule nuclei shows that the nucleoplasmic Hrb87F proteins also forms “trail”-like structures, which fully overlap (yellow) with the hsrω-n ncRNA signal. DAPI stained DNA is shown in blue. Arrowheads denote the 93D cytologenetic region in wild type.
(TIF)

Figure S5 *ISWI* omega “trails” are not a fixation artifact. (A) Live larval Malpighian tubule whole nucleus expressing the Squid-GFP fusion protein-trap allele (*Squid^GFP^*) [16] showing the presence of the Squid protein in typical omega speckles. (B) Live *ISWI^1^/ISWI^2^* mutant Malpighian tubule whole nucleus expressing Squid-GFP [16] protein (*ISWI; Squid^GFP^*) shows the presence of omega trails. Arrowheads point to the 93D cytologenetic region.
(TIF)

Figure S6 *ISWI* mutant omega trails are not due to chromosome decondensation *per se*. (A, B and C) DAPI staining of *jil^1^*, *ada2* and *gcn5* homozygous mutant salivary gland (SG) polytene chromosomes, respectively, highlights various types of chromosome organization and condensation defects [17], [18] that are reminiscent of those present in the *ISWI* null polytene nuclei (Figure 1C) [3], [4]. DAPI stained DNA is shown in gray. Asterisks indicate the “puffed” male X chromosome. (D, E and F) FRISH on homozygous *jil^1^*, *ada2* or *gcn5* mutant Malpighian tubule nuclei using the 280b tandem repeat unit riboprobe to detect the hsrω-n ncRNA (green) does not show any “trail”-like structures seen in the *ISWI* mutant nuclei (Figure 2B). DAPI stained DNA is shown in blue. Arrowheads denote the 93D cytologenetic location.
(TIF)

Figure S7 Loss of ISWI does not alter levels of hsrω transcripts or of omega speckles associated hnRNPs. (A) RT-PCR analysis on total RNA extracted from wild type (*w^1118^*) and *ISWI* null (*ISWI^1^/ISWI^2^*) Malpighian tubules using primers specific for the 280b repeat unit of hsrω-n or the Act5C transcripts (Text S1). The level of PCR amplified signals relative (in percentage) to that in *w^1118^* is shown at the right of each row. (B) Western blot of Malpighian tubule nuclear extracts [35] from wild type (*w^1118^*) and *ISWI* null (*ISWI^1^/ISWI^2^*) mutant larvae challenged with ISWI (anti-ISWI) [4], Hrb87F (anti-Hrb87F), PEP (anti-PEP), Sqd (anti-Sqd) or Tubulin (anti-Tub; SIGMA) antibodies. Quantification of the Western blot signals relative to *w^1118^* extract (in percentage) is shown to the right of each panel.
(TIF)

Figure S8 Splicing of hsrω transcript and polyA^+^ RNA export are not affected by loss of *ISWI* function. (A) Schematic representation of the ∼10 kb *hsrω* gene structure. The hsrω-n ncRNA corresponds to this entire region, including the 700 bp intron region [19] and is believed to be responsible for organization of the omega speckles [14]. Recently, it has been found that the omega speckle associated hsrω-n ncRNA exists in unspliced as well as spliced forms [20], which can be easily distinguished by RT-PCR because they produce distinct amplicons differing by 700 bp. (B) RT-PCR on total RNA extracted from *w^1118^* (wild type) and *ISWI* null (*ISWI^1^/ISWI^2^*) mutant salivary glands (SG) and Malpighian tubules (MT) was conducted using primers that amplify the Act5C mRNA and that can distinguish between the unspliced and spliced hsrω-n transcripts. The RT-PCR products are identical in wild type and *ISWI* null backgrounds. Arrows indicate the 1.8 Kb unspliced and the 1.1 Kb spliced PCR products (see Text S1 for primer sequences). (C) Northern blot of total RNA extracted from *w^1118^* (wild type) and *ISWI* null (*ISWI^1^/ISWI^2^*) mutant salivary glands (SG) and Malpighian tubules (MT) hybridized with the 280b tandem repeat unit probe specific for the hsrω-n ncRNA. Hybridization with probe for the housekeeping GAPDH mRNA was used as the RNA loading control. Note the absence of any differences between amplicons or the hsrω-n RNA size in Northern blot between wild type and *ISWI*-null backgrounds. (D) FRISH on *w^1118^* (wild type) and *ISWI*-null (*ISWI^1^/ISWI^2^*) mutant salivary glands using an Oligo-dT probe directly labeled with Cy3. The insets (upper left) show higher magnification images of the Oligo-dT signals corresponding to the white boxed areas. Note the comparable hybridization signal in wild type and *ISWI*-null backgrounds. DAPI stained DNA is shown in blue while Oligo-dT hybridization signal is in red.
(TIF)

Figure S9 Organization of omega speckles in *ISWI* mutants is affected under heat-shock conditions. FRISH on (A) *w^1118^* (wild type), (B) *ISWI^1^/ISWI^2^* mutant (*ISWI*) Malpighian tubule whole nuclei under control condition (Control), using the hsrω-n RNA specific 280b tandem repeat unit riboprobe (green) showing the fine nucleoplasmic omega speckles close to chromatin areas (see double arrow in magnified image in the inset) [13], [14] in A or as “trails” in B. (C) FRISH against hsrω-n RNA (green) on *w^1118^* and (D) on *ISWI* mutant Malpighian tubule whole nuclei after heat shock (Heat Shock). DNA was counterstained with DAPI (blue). The insets show higher magnification images of DAPI and hsrω signals corresponding to the white boxed areas. The double arrows point at some representative examples of omega speckles present under control or after heat shock conditions in wild type and *ISWI* mutant cells. Arrowheads point to the 93D cytogenetic region. Under conditions of heat shock, the hsrω-n RNA binding proteins are released from their chromosomal locations and are quickly sequestered by the concomitantly elevated levels of hsrω-n transcripts [13], [14]. With increasing levels of sequestration, the omega speckles themselves coalesce (see double arrow under heath shock condition), initially forming larger nucleoplasmic clusters and finally, all the nuclear hsrω-n ncRNA and the associated proteins get restricted to the *hsrω* gene locus at the 93D cytogenetic location (see arrowhead under heat shock condition, C and D). As noted earlier (Figure 2B, Figures S4 and S5), the omega speckle associated ncRNA and proteins show “trail”-like organization in *ISWI*-null cells under control conditions and this is also seen after heat shock. Interestingly, the number of coalesced omega “trails” in *ISWI* mutant heat shock Malpighian tubule nuclei is fewer than in wild type.
(TIF)

Figure S10 *ISWI* mutant omega trails are present in polytene as well as in diploid nuclei. (A) Immuno-FRISH for ISWI (red) and hsrω-n RNA (green) on *ISWI^1^/ISWI^2^* mutant (*ISWI*) salivary gland (SG) squashed nucleus (DAPI stained chromatin is in blue) shows presence of omega “trails” adjacent to polytene chromosomes. (B) Immuno-FRISH on *ISWI* mutant larval salivary glands co-expressing *ey-GAL4* driven *hsrω-RNAi^3^* transgene reveals disappearance of omega “trails” following the reduced hsrω-n RNA; under this condition the only signal seen after FRISH is the one present at the 93D cytological location where the *hsrω* gene continues to transcribe. The very low ISWI staining (red) seen on *ISWI*-null nuclei is due to the maternal contribution [3]. The asterisks indicate the male X chromosome while the arrowheads point to the 93D cytogenetic region. (C) FRISH for hsrω-n RNA on wild type (*w^1118^*) diploid cells from larval testis also shows the presence of classic omega speckles. (D) The *ISWI^1^/ISWI^2^* mutant (*ISWI*) larval testis cells show the omega “trail” structures, similar to those seen in salivary glands and Malpighian tubules from *ISWI*-null cells, though smaller in size. This indicates that omega speckles disorganization is a general nuclear defect following loss of *ISWI* function.
(TIF)

Figure S11 ISWI and omega speckle associated hnRNPs show partial overlap. (A) Double immunostaining for the ISWI (green) and NonA (red) proteins on *w^1118^* salivary gland (SG) squashed nuclei, shows little overlap between the two proteins on polytene chromosomes. One representative example of the few sites that exhibit overlapping distribution of the two proteins is indicated by white arrow. (B) Double immunostaining for the ISWI (green) and Hrb87F (red) proteins on wild type (*w^1118^*) salivary gland (SG) squashed nuclei; in this case also there is little overlap between the two proteins on polytene chromosomes. One of the few sites where both the proteins are present is indicated by white arrow. (C) Confocal sections showing double immunostaining for ISWI and NonA proteins on wild type (*w^1118^*) Malpighian tubule (MT) whole nuclei highlights the presence of some sites of partial overlap between ISWI (green) and NonA (red) proteins. (D) Double immunostained confocal section of wild type (*w^1118^*) Malpighian tubule (MT) whole nuclei also shows sites where ISWI (green) and Hrb87F (red) proteins display partial overlap.
(TIF)

Figure S12 Predicted secondary structure of hsrω ncRNA 280 bp repeat unit. Secondary structure prediction of the 280b hsrω-n ncRNA repeat unit used for the binding and ATPase assays (Figure 5). Secondary structure prediction was obtained using the RNA secondary structure prediction tool present at the GeneBee molecular biology server (http://www.genebee.msu.su/services/rna2_reduced.html).
(TIF)

Figure S13 Effect of hsrω encoding DNA and of nucleosomes on hsrω-stimulated ISWI ATPase activity. (A) Stimulation of the ATPase activity of ISWI by generic plasmid DNA, linear double stranded 280 bp DNA encoding the hsrω-n repeat unit or *in vitro* transcribed 280b repeat unit RNA was assayed *in vitro* by thin layer chromatography in the presence of radioactive ATP-γ^33P^. Samples were incubated for 30 minutes. (B) Effect of nucleosome presence on the hsrω-stimulated ISWI ATPase activity. Samples were incubated for 60 minutes. When nucleosomes (Nucl) and hsrω ncRNA were combined in the same reaction, the amount of each was half of the amount used to test them alone. Stimulation of the ATPase activity by the used nucleic acids assayed is noted below each lane as percentage of the ATP hydrolysed (P^33^ = P^33^ radioactive labeled hydrolyzed gamma phosphate, AMP-P-P^33^ = radioactive labeled non-hydrolyzed ATP).
(TIF)

Table S1 (A) Results of the genetic interaction test between *hsrω* and *sqd* alleles with *ISWI*, as revealed by the *ISWI^EGUF^* eye test (see also Figure S1 and Text S1). The nature of the alleles tested has been obtained from the Flybase (www.flybase.org); a – sign in column 2 indicates that nature of the allele is not know. (B) RNAi based reduction of hsrω transcripts using either the *ey-GAL4* or the *Act5C-GAL4* driver prolongs the survival of *ISWI* trans-heterozygous null mutants to pupal stage (also see Figure 1K), though none of these pupae enclose as flies. n = number of larvae scored for each genotype.
(DOC)

Text S1 Supporting Materials and Methods.
(DOC)

Video S1 The composite movie shows time lapse animations of live Malpighian tubule (first 3 movies) or diploid gonadal (fourth movie) cells, showing rapid movements of green fluorescent omega speckles (using the omega speckle associated Hrb87F-GFP or Sqd-GFP hnRNPs). Chromatin is seen in red (in clips 2–4) because of a transgene expressing the H2A-RFP fusion histone protein. Time lapse movies have been acquired for 2–5 minutes. Note the very rapid movements of omega speckles in nucleoplasm in polytene as well as diploid nuclei.
(AVI)

Video S2 An animation model to explain how *ISWI* phenotypes (eye development and chromosome condensation defects) are suppressed by hsrω-RNAi and how, in turn, the *ISWI* null condition results in omega ‘trail’ phenotype.
(MOV)
