# Supplementary figures and images for: The ISWI Chromatin Remodeler Organizes the hsrω ncRNA–Containing Omega Speckle Nuclear Compartments
Source: PLoS Genet. 2011 May 26;7(5):e1002096. doi: 10.1371/journal.pgen.1002096 (PMC3102753; doi:10.1371/journal.pgen.1002096)

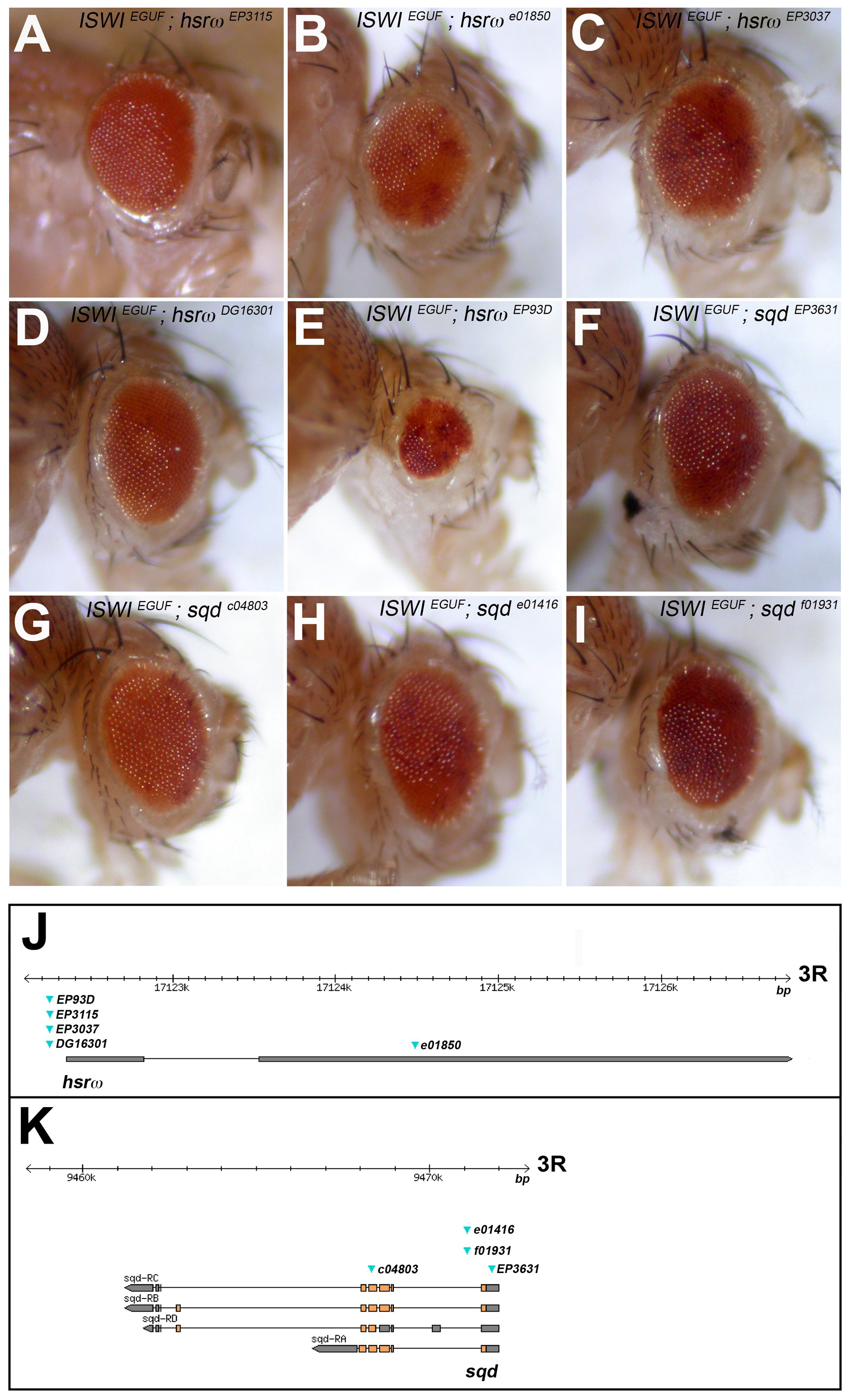

Supplement: Figure S1 — Genetic interactions between ISWI, hsrω and sqd. (A, B, C and D) Eye phenotypes resulting from eye homozygous for the ISWI2 allele (ISWIEGUF) [11] are suppressed by the hsrω alleles, hsrωEP3115 [10], hsrωe01850, hsrω EP3037 and hsrω DG16301, respectively, thus reconfirming the genetic interaction between ISWI and hsrω as reported earlier [10] (also see Table S1A). (E) ey-GAL4 directed over-expression of hsrω through the hsrωEP93D allele [15] enhances ISWIEGUF eye phenotype, suggesting that an excess of hsrω transcripts antagonizes ISWI function (also see Table S1A). (F, G, H and I) ISWIEGUF eye defects are suppressed by the sqdEP3631, sqdc04803, sqd e01416 and sqd f01931 alleles, thus revalidating a genetic interaction between ISWI and sqd [10] (also see Table S1A). (J and K) Schematic genetic map showing locations of the hsrω and sqd insertion alleles, respectively, used in the above ISWIEGUF assay. Introns are displayed as thin lines, exons by filled boxes. Noncoding regions are in grey and the coding parts are shown in orange. Alternatively spliced transcripts of the sqd gene, as reported on the FlyBase (www.flybase.org), are also shown. (TIF) [file pgen.1002096.s001.tif]

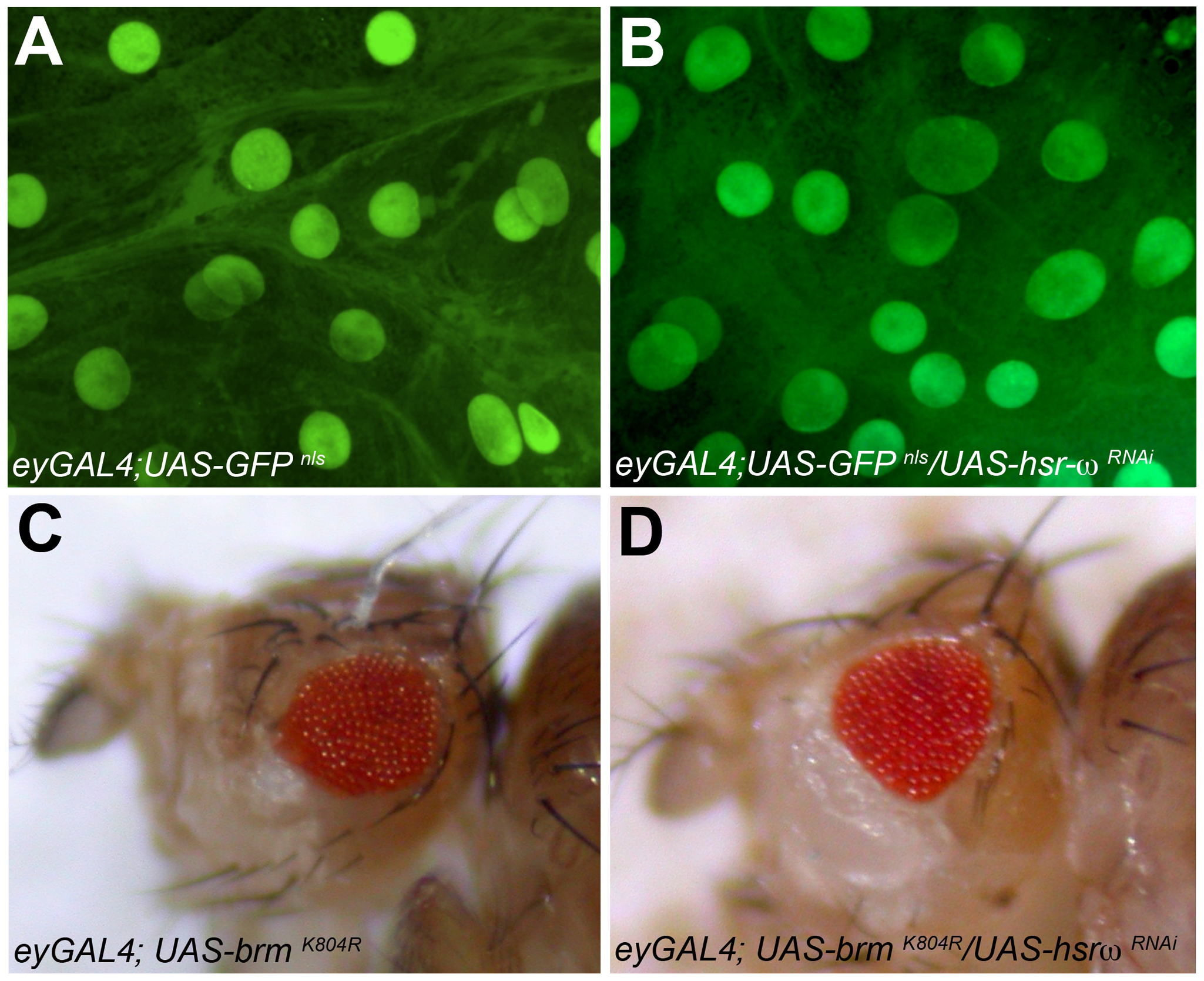

Supplement: Figure S2 — The suppression of ISWI phenotype by hsrω-RNAi is highly specific. (A and B) Nuclear expression of GFP from a UAS-GFPnls transgene is not reduced when the hsrω ncRNA is knocked down by RNAi, indicating that loss of hsrω function does not interfere with the GAL4/UAS driving system, as also previously shown [15], [26]. (C) The brm gene encodes a chromatin remodeler but with functions opposing ISWI [16]. Eye specific mis-expression of the catalytically inactive brmK804R allele produces rough and reduced eyes that are reminiscent of those obtained with the catalytically inactive ISWIK159R allele. (D) Unlike the suppression of ISWIK159R phenotype (see Figure 1E–1H), eye-specific expression of hsrω-RNAi does not suppress brmK804R eye defects, strongly indicating that the suppression of ISWIK159R defects by hsrω-RNAi is specific. (TIF) [file pgen.1002096.s002.tif]

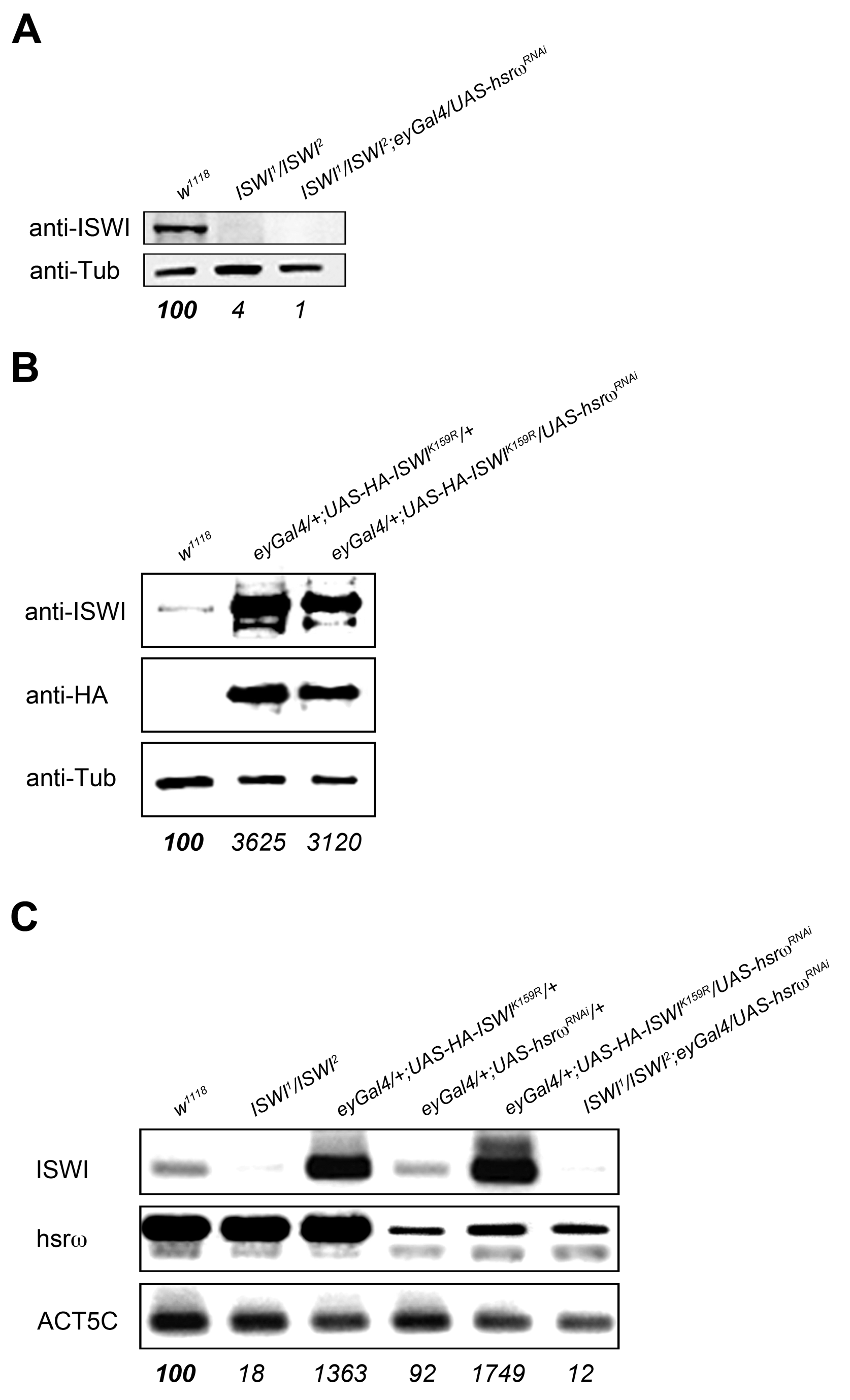

Supplement: Figure S3 — The hsrω-RNAi does not cause any change in ISWI protein as well as mRNA stability. (A) Western blots of salivary gland nuclear extracts [35] from wild type (w1118), ISWI null (ISWI1/ISWI2), and ISWI; hsrω double mutants (ISWI1/ISWI2; ey-Gal4/UAS-hsrω-RNAi 3) challenged with ISWI (anti-ISWI) [4] and Tubulin (anti-Tub; SIGMA) antibodies. The level of ISWI positive signal relative to w1118 extract (as percentage) is noted below each lane. (B) Western blot of salivary gland nuclear extracts [35] derived from w1118, HA-tagged ISWIK159R mis-expressing mutants (ey-Gal4/+; UAS-HA-ISWI K159R /+), or form glands that co-express, HA-tagged ISWIK159R and hsrω-RNAi (ey-Gal4/+; UAS-HA-ISWI K159R/UAS-hsrωRNAi 3) transgenes challenged with ISWI (anti-ISWI) [4], HA epitope (anti-HA; ROCHE) or Tubulin (anti-Tub; SIGMA) antibodies. Level of ISWI positive signal relative to w1118 extract (as percentage) is noted below each lane. (C) RT-PCR analysis of total RNA extracted from salivary glands derived from w1118, ISWI-null (ISWI1/ISWI2), HA-tagged ISWIK159R expressing mutants alone (ey-GAL4/+; UAS-HA-ISWI K159R /+), hsrω knock down alone (ey-GAL4/+; UAS-hsrωRNAi3/+), or glands co-expressing HA-tagged ISWIK159R and hsrω-RNAi (eyGal4/+; UAS-HA-ISWI K159R/UAS-hsrωRNAi3) and finally from glands that are ISWI; hsrω double mutants (ISWI1/ISWI2; ey-GAL4/UAS-hsrωRNAi 3) using primers specific for ISWI, hsrω-n or the Act5C transcripts. The quantification of PCR amplified ISWI mRNA signal relative to wild type (w1118) extract is shown in percentage below each lane. (TIF) [file pgen.1002096.s003.tif]

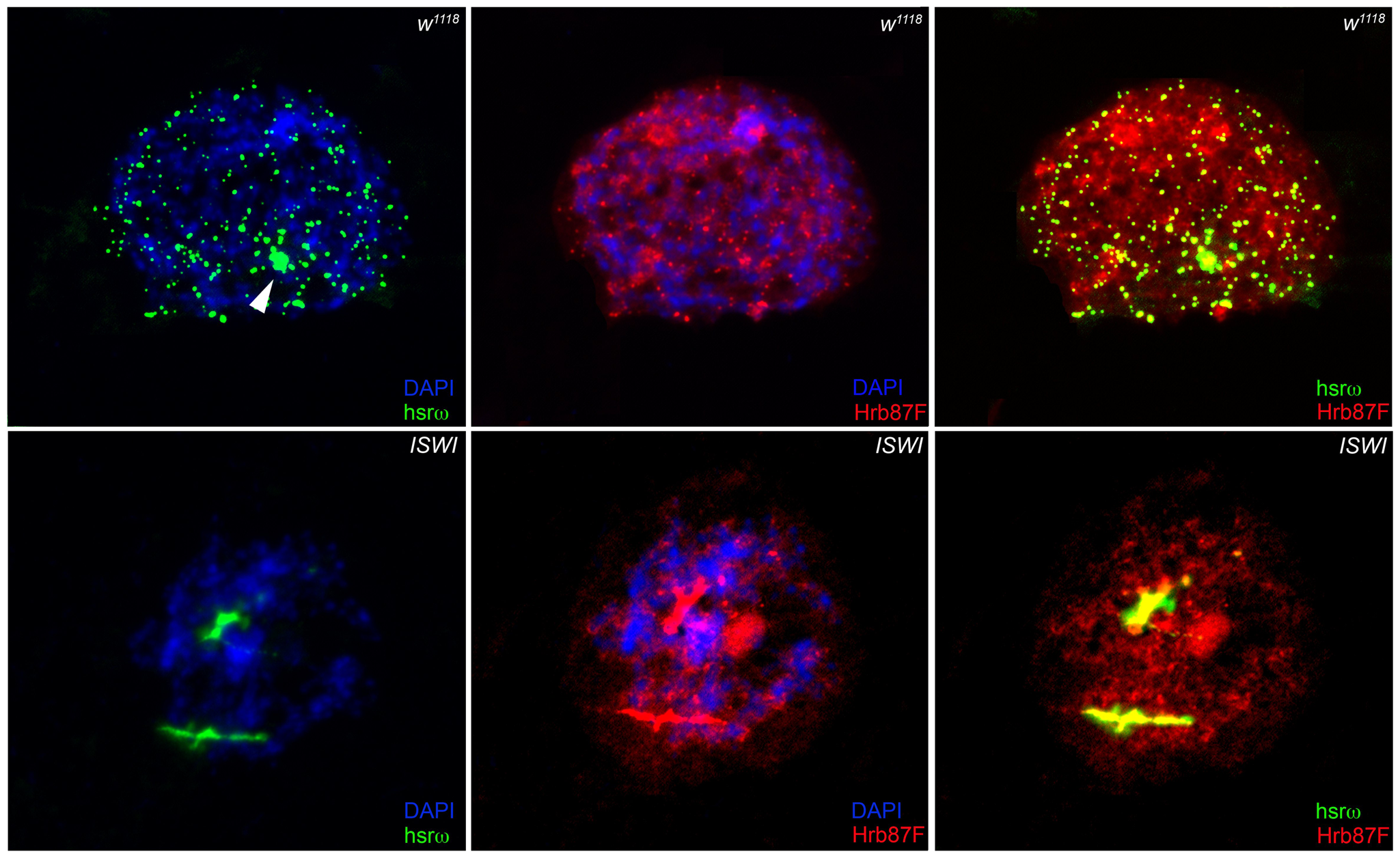

Supplement: Figure S4 — Hrb87F forms omega trails in ISWI mutant nuclei. The Hrb87F protein also co-localizes in the nucleoplasm with the hsrω-n ncRNA in the nucleoplasmic omega speckles [14] as shown by immunostaining for Hrb87F (red) combined with FRISH for hsrω-n (green) ncRNA on intact wild type (w1118) Malpighian tubule nuclei. Immuno-FRISH of Hrb87F (red) and hsrω-n ncRNA (green) on intact ISWI1/ISWI2 (ISWI) Malpighian tubule nuclei shows that the nucleoplasmic Hrb87F proteins also forms “trail”-like structures, which fully overlap (yellow) with the hsrω-n ncRNA signal. DAPI stained DNA is shown in blue. Arrowheads denote the 93D cytologenetic region in wild type. (TIF) [file pgen.1002096.s004.tif]

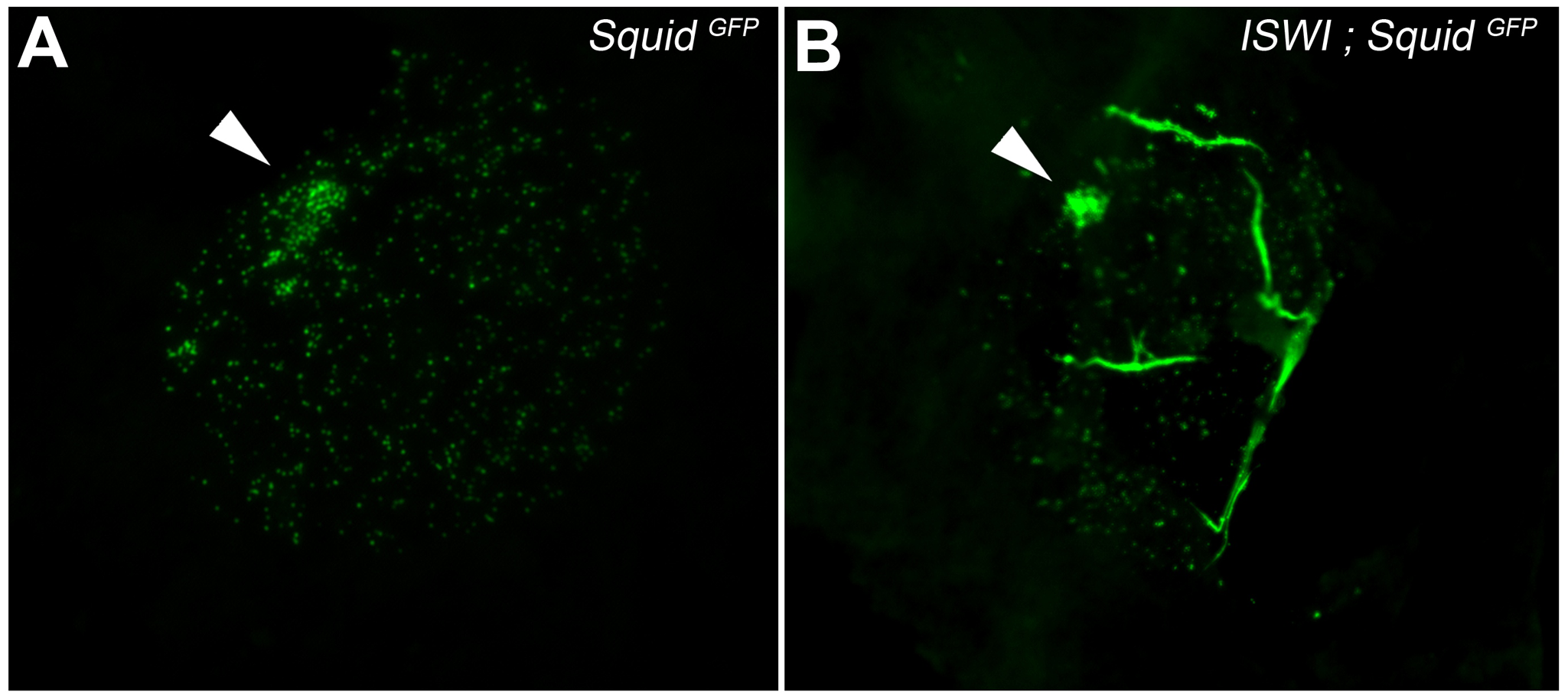

Supplement: Figure S5 — ISWI omega “trails” are not a fixation artifact. (A) Live larval Malpighian tubule whole nucleus expressing the Squid-GFP fusion protein-trap allele (SquidGFP) [16] showing the presence of the Squid protein in typical omega speckles. (B) Live ISWI1/ISWI2 mutant Malpighian tubule whole nucleus expressing Squid-GFP [16] protein (ISWI; SquidGFP) shows the presence of omega trails. Arrowheads point to the 93D cytologenetic region. (TIF) [file pgen.1002096.s005.tif]

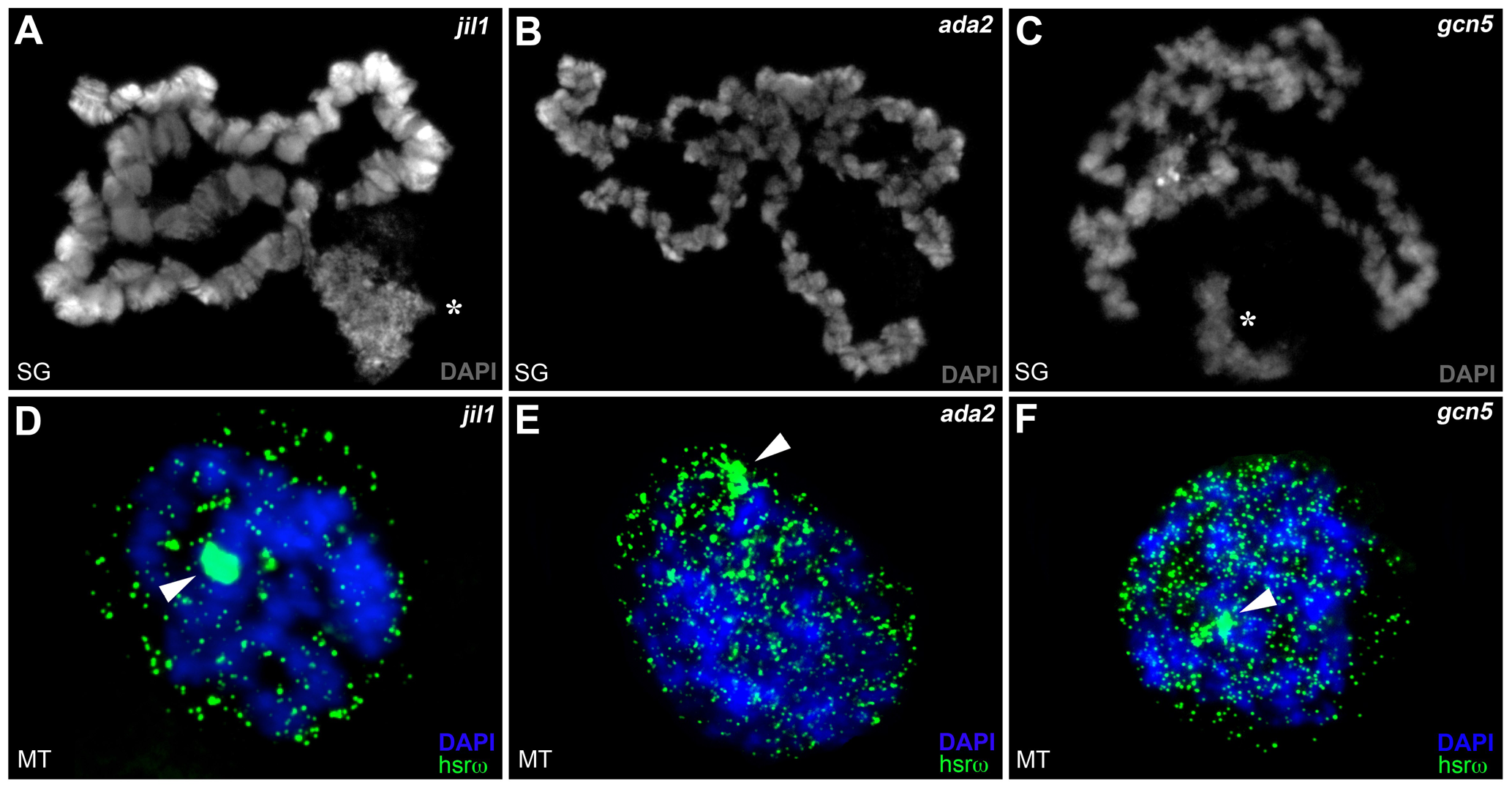

Supplement: Figure S6 — ISWI mutant omega trails are not due to chromosome decondensation per se. (A, B and C) DAPI staining of jil1, ada2 and gcn5 homozygous mutant salivary gland (SG) polytene chromosomes, respectively, highlights various types of chromosome organization and condensation defects [17], [18] that are reminiscent of those present in the ISWI null polytene nuclei (Figure 1C) [3], [4]. DAPI stained DNA is shown in gray. Asterisks indicate the “puffed” male X chromosome. (D, E and F) FRISH on homozygous jil1, ada2 or gcn5 mutant Malpighian tubule nuclei using the 280b tandem repeat unit riboprobe to detect the hsrω-n ncRNA (green) does not show any “trail”-like structures seen in the ISWI mutant nuclei (Figure 2B). DAPI stained DNA is shown in blue. Arrowheads denote the 93D cytologenetic location. (TIF) [file pgen.1002096.s006.tif]

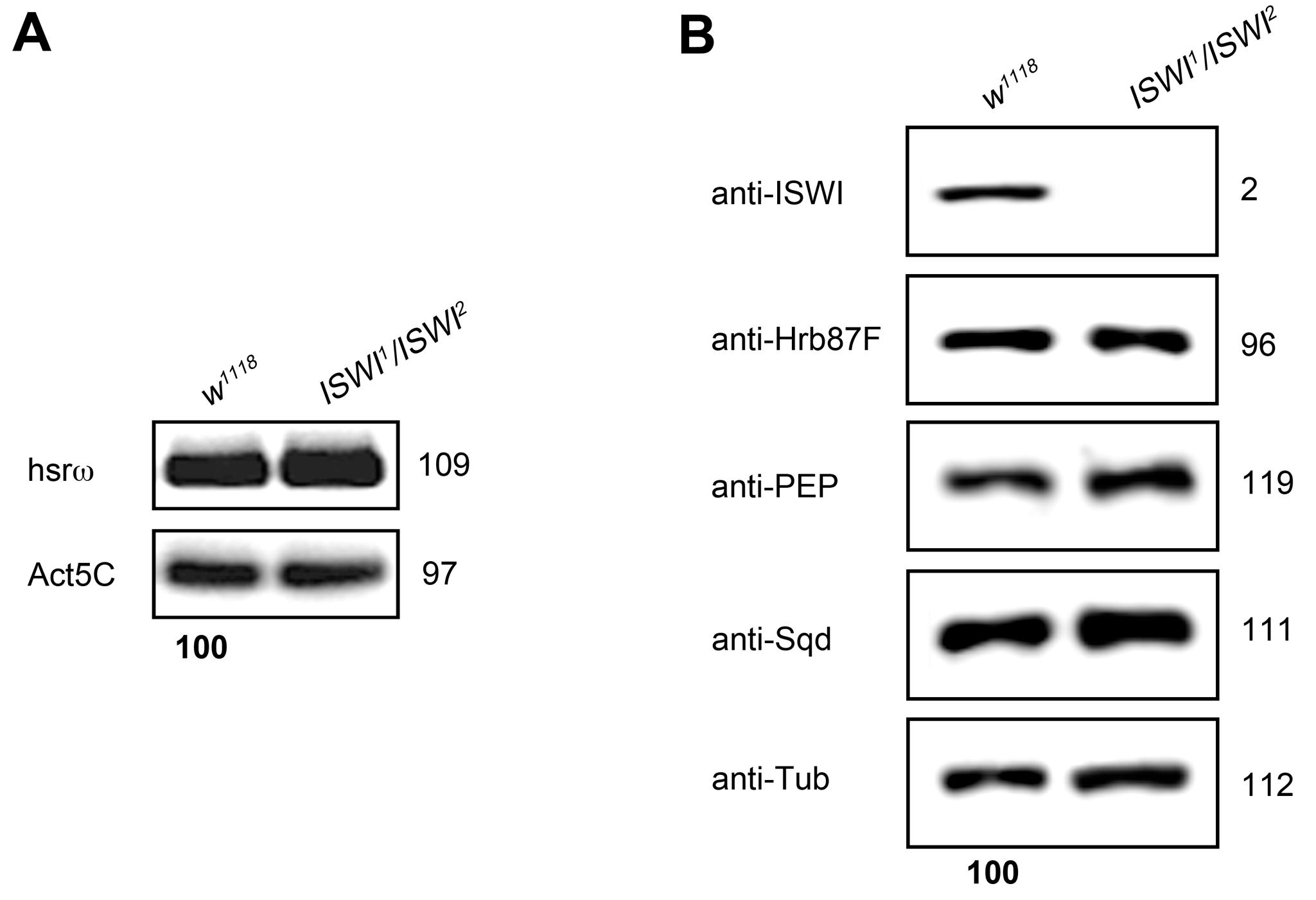

Supplement: Figure S7 — Loss of ISWI does not alter levels of hsrω transcripts or of omega speckles associated hnRNPs. (A) RT-PCR analysis on total RNA extracted from wild type (w1118) and ISWI null (ISWI1/ISWI2) Malpighian tubules using primers specific for the 280b repeat unit of hsrω-n or the Act5C transcripts (Text S1). The level of PCR amplified signals relative (in percentage) to that in w1118 is shown at the right of each row. (B) Western blot of Malpighian tubule nuclear extracts [35] from wild type (w1118) and ISWI null (ISWI1/ISWI2) mutant larvae challenged with ISWI (anti-ISWI) [4], Hrb87F (anti-Hrb87F), PEP (anti-PEP), Sqd (anti-Sqd) or Tubulin (anti-Tub; SIGMA) antibodies. Quantification of the Western blot signals relative to w1118 extract (in percentage) is shown to the right of each panel. (TIF) [file pgen.1002096.s007.tif]

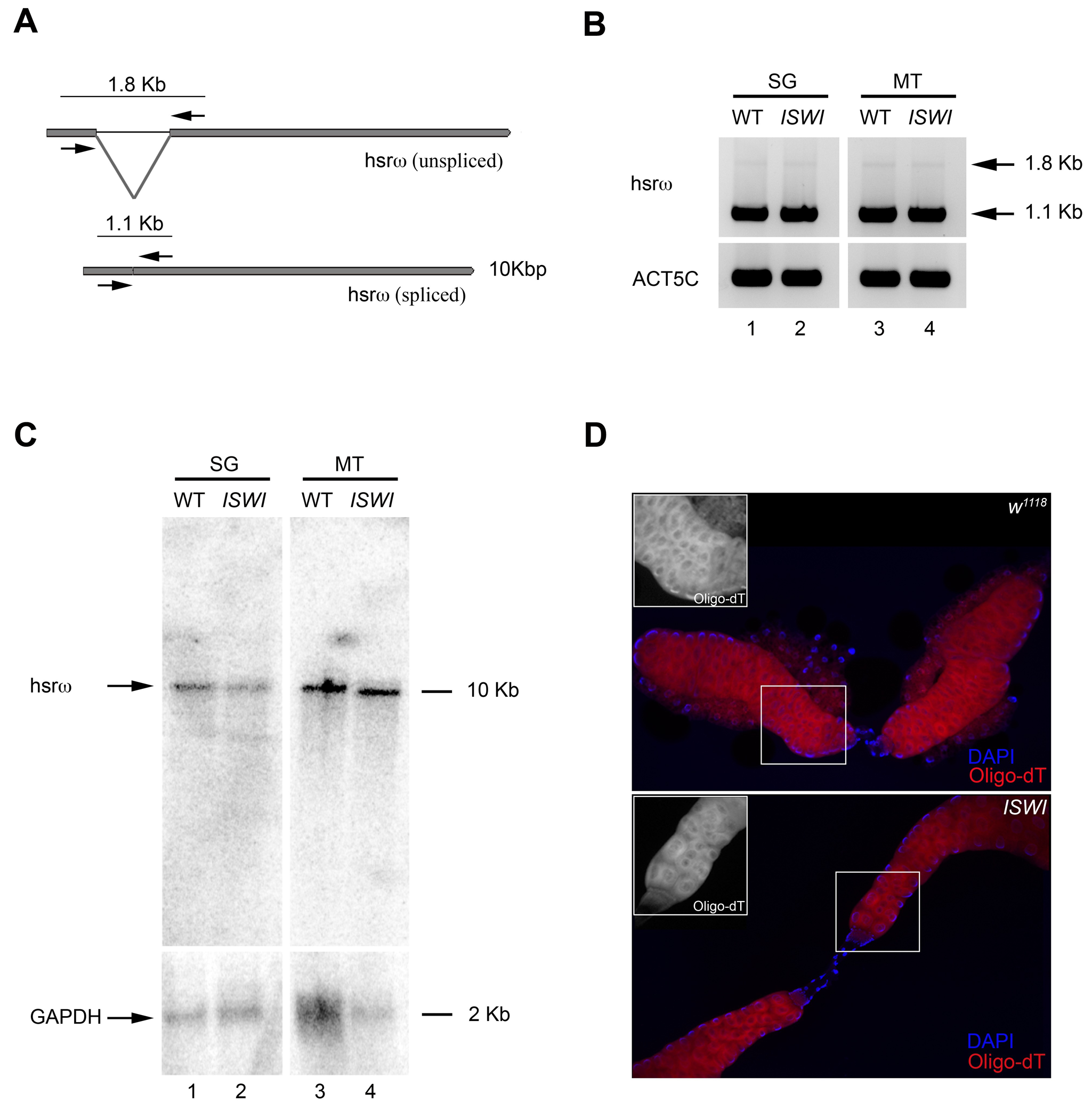

Supplement: Figure S8 — Splicing of hsrω transcript and polyA+ RNA export are not affected by loss of ISWI function. (A) Schematic representation of the ∼10 kb hsrω gene structure. The hsrω-n ncRNA corresponds to this entire region, including the 700 bp intron region [19] and is believed to be responsible for organization of the omega speckles [14]. Recently, it has been found that the omega speckle associated hsrω-n ncRNA exists in unspliced as well as spliced forms [20], which can be easily distinguished by RT-PCR because they produce distinct amplicons differing by 700 bp. (B) RT-PCR on total RNA extracted from w1118 (wild type) and ISWI null (ISWI1/ISWI2) mutant salivary glands (SG) and Malpighian tubules (MT) was conducted using primers that amplify the Act5C mRNA and that can distinguish between the unspliced and spliced hsrω-n transcripts. The RT-PCR products are identical in wild type and ISWI null backgrounds. Arrows indicate the 1.8 Kb unspliced and the 1.1 Kb spliced PCR products (see Text S1 for primer sequences). (C) Northern blot of total RNA extracted from w1118 (wild type) and ISWI null (ISWI1/ISWI2) mutant salivary glands (SG) and Malpighian tubules (MT) hybridized with the 280b tandem repeat unit probe specific for the hsrω-n ncRNA. Hybridization with probe for the housekeeping GAPDH mRNA was used as the RNA loading control. Note the absence of any differences between amplicons or the hsrω-n RNA size in Northern blot between wild type and ISWI-null backgrounds. (D) FRISH on w1118 (wild type) and ISWI-null (ISWI1/ISWI2) mutant salivary glands using an Oligo-dT probe directly labeled with Cy3. The insets (upper left) show higher magnification images of the Oligo-dT signals corresponding to the white boxed areas. Note the comparable hybridization signal in wild type and ISWI-null backgrounds. DAPI stained DNA is shown in blue while Oligo-dT hybridization signal is in red. (TIF) [file pgen.1002096.s008.tif]

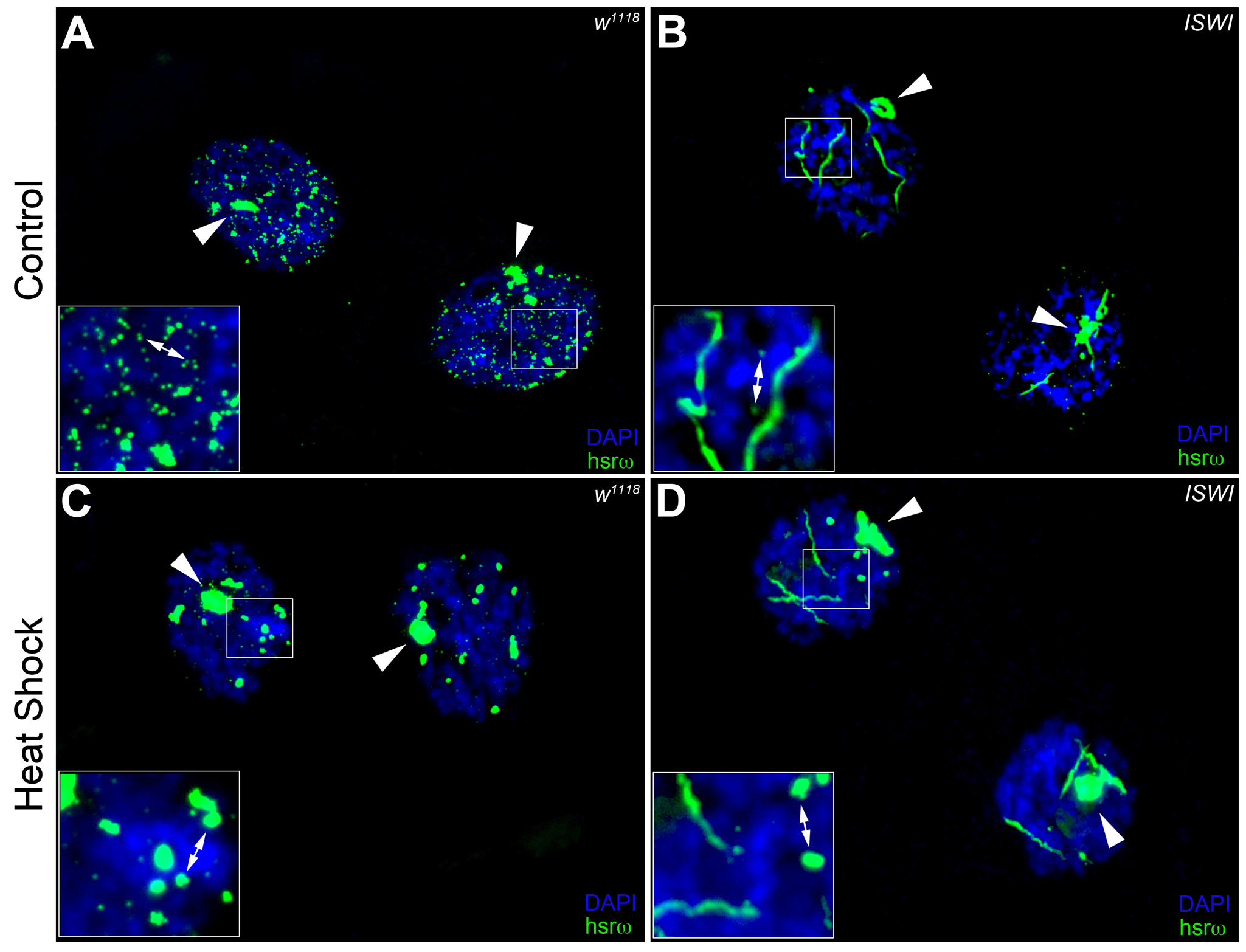

Supplement: Figure S9 — Organization of omega speckles in ISWI mutants is affected under heat-shock conditions. FRISH on (A) w1118 (wild type), (B) ISWI1/ISWI2 mutant (ISWI) Malpighian tubule whole nuclei under control condition (Control), using the hsrω-n RNA specific 280b tandem repeat unit riboprobe (green) showing the fine nucleoplasmic omega speckles close to chromatin areas (see double arrow in magnified image in the inset) [13], [14] in A or as “trails” in B. (C) FRISH against hsrω-n RNA (green) on w1118 and (D) on ISWI mutant Malpighian tubule whole nuclei after heat shock (Heat Shock). DNA was counterstained with DAPI (blue). The insets show higher magnification images of DAPI and hsrω signals corresponding to the white boxed areas. The double arrows point at some representative examples of omega speckles present under control or after heat shock conditions in wild type and ISWI mutant cells. Arrowheads point to the 93D cytogenetic region. Under conditions of heat shock, the hsrω-n RNA binding proteins are released from their chromosomal locations and are quickly sequestered by the concomitantly elevated levels of hsrω-n transcripts [13], [14]. With increasing levels of sequestration, the omega speckles themselves coalesce (see double arrow under heath shock condition), initially forming larger nucleoplasmic clusters and finally, all the nuclear hsrω-n ncRNA and the associated proteins get restricted to the hsrω gene locus at the 93D cytogenetic location (see arrowhead under heat shock condition, C and D). As noted earlier (Figure 2B, Figures S4 and S5), the omega speckle associated ncRNA and proteins show “trail”-like organization in ISWI-null cells under control conditions and this is also seen after heat shock. Interestingly, the number of coalesced omega “trails” in ISWI mutant heat shock Malpighian tubule nuclei is fewer than in wild type. (TIF) [file pgen.1002096.s009.tif]

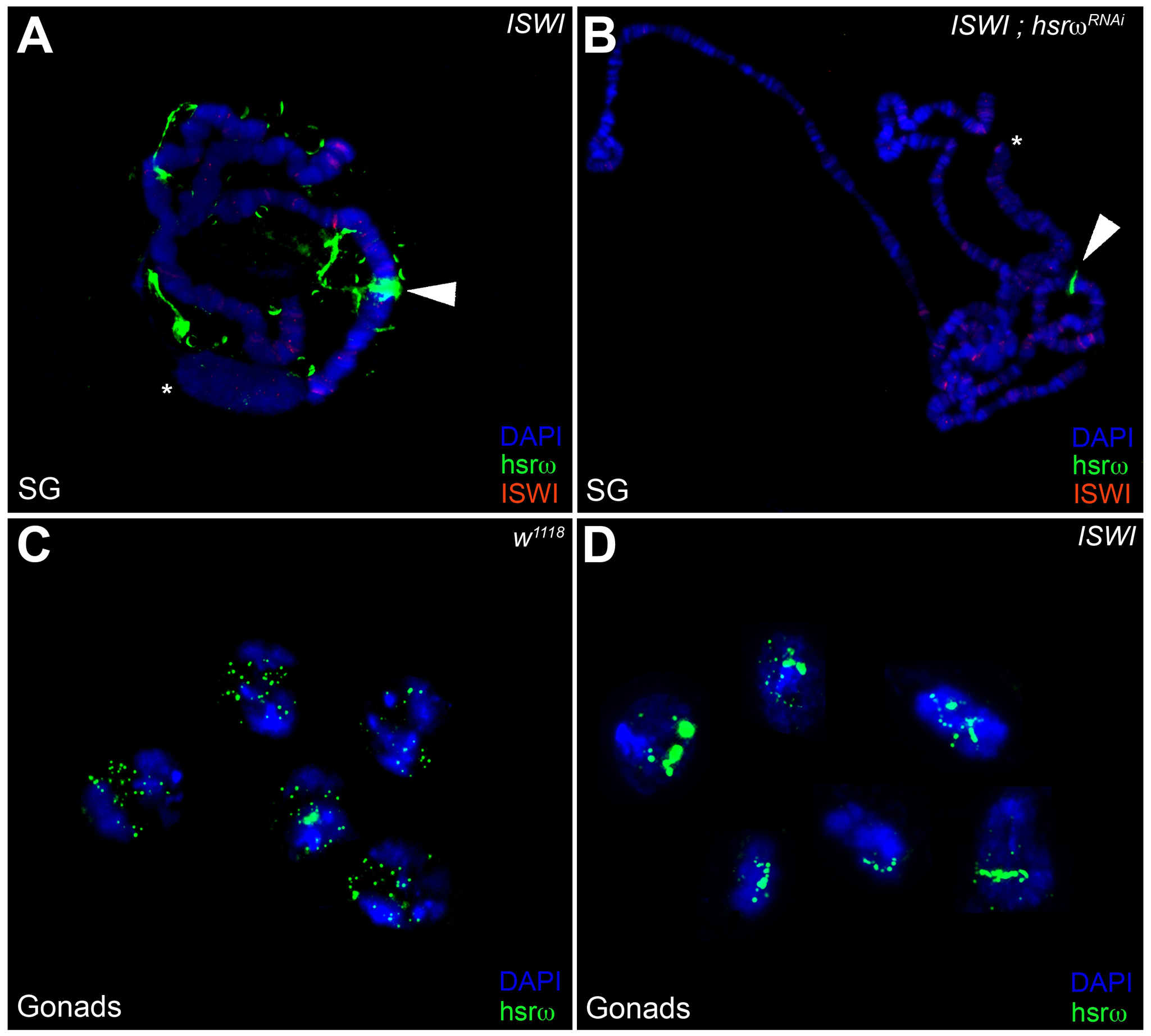

Supplement: Figure S10 — ISWI mutant omega trails are present in polytene as well as in diploid nuclei. (A) Immuno-FRISH for ISWI (red) and hsrω-n RNA (green) on ISWI1/ISWI2 mutant (ISWI) salivary gland (SG) squashed nucleus (DAPI stained chromatin is in blue) shows presence of omega “trails” adjacent to polytene chromosomes. (B) Immuno-FRISH on ISWI mutant larval salivary glands co-expressing ey-GAL4 driven hsrω-RNAi3 transgene reveals disappearance of omega “trails” following the reduced hsrω-n RNA; under this condition the only signal seen after FRISH is the one present at the 93D cytological location where the hsrω gene continues to transcribe. The very low ISWI staining (red) seen on ISWI-null nuclei is due to the maternal contribution [3]. The asterisks indicate the male X chromosome while the arrowheads point to the 93D cytogenetic region. (C) FRISH for hsrω-n RNA on wild type (w1118) diploid cells from larval testis also shows the presence of classic omega speckles. (D) The ISWI1/ISWI2 mutant (ISWI) larval testis cells show the omega “trail” structures, similar to those seen in salivary glands and Malpighian tubules from ISWI-null cells, though smaller in size. This indicates that omega speckles disorganization is a general nuclear defect following loss of ISWI function. (TIF) [file pgen.1002096.s010.tif]

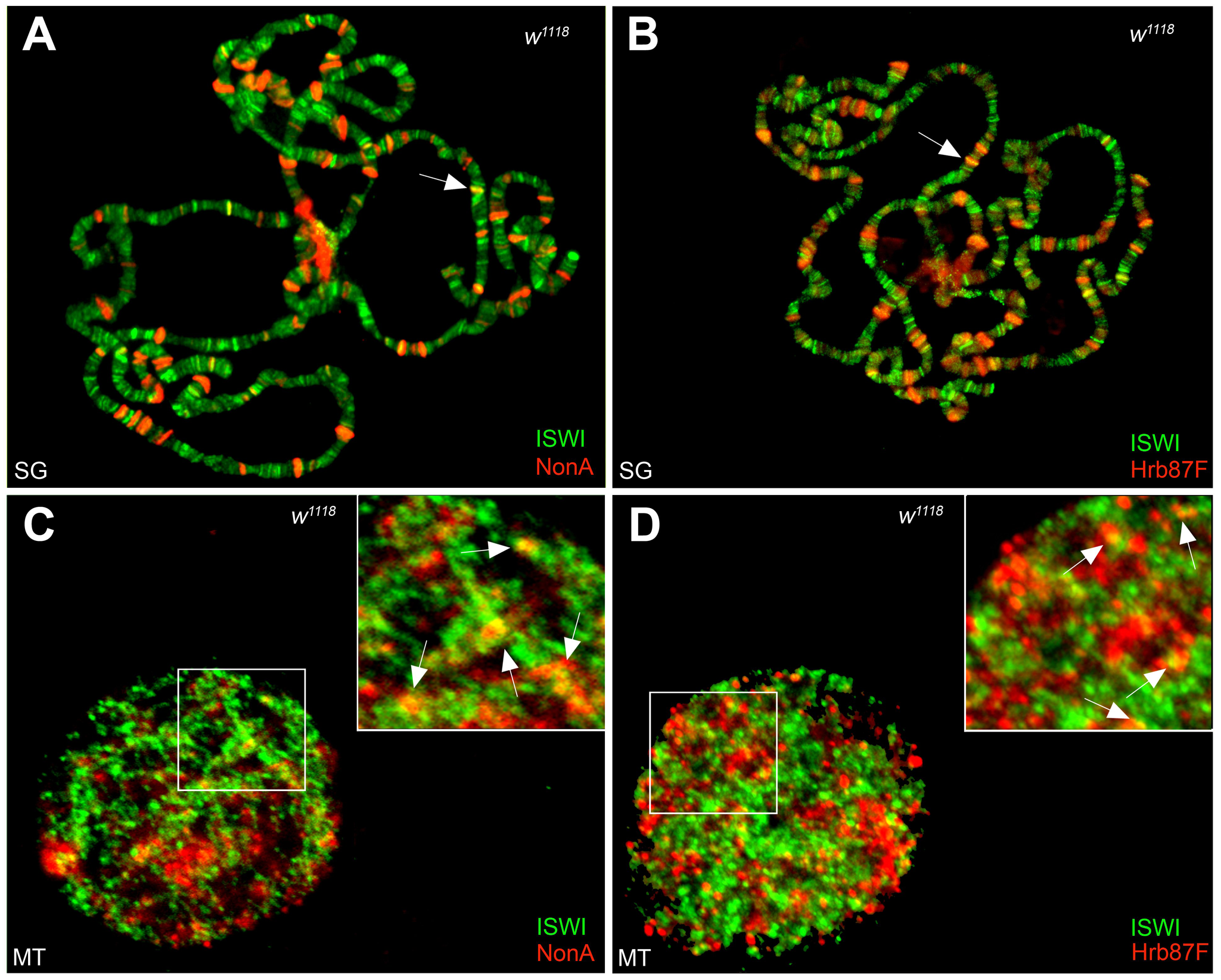

Supplement: Figure S11 — ISWI and omega speckle associated hnRNPs show partial overlap. (A) Double immunostaining for the ISWI (green) and NonA (red) proteins on w1118 salivary gland (SG) squashed nuclei, shows little overlap between the two proteins on polytene chromosomes. One representative example of the few sites that exhibit overlapping distribution of the two proteins is indicated by white arrow. (B) Double immunostaining for the ISWI (green) and Hrb87F (red) proteins on wild type (w1118) salivary gland (SG) squashed nuclei; in this case also there is little overlap between the two proteins on polytene chromosomes. One of the few sites where both the proteins are present is indicated by white arrow. (C) Confocal sections showing double immunostaining for ISWI and NonA proteins on wild type (w1118) Malpighian tubule (MT) whole nuclei highlights the presence of some sites of partial overlap between ISWI (green) and NonA (red) proteins. (D) Double immunostained confocal section of wild type (w1118) Malpighian tubule (MT) whole nuclei also shows sites where ISWI (green) and Hrb87F (red) proteins display partial overlap. (TIF) [file pgen.1002096.s011.tif]

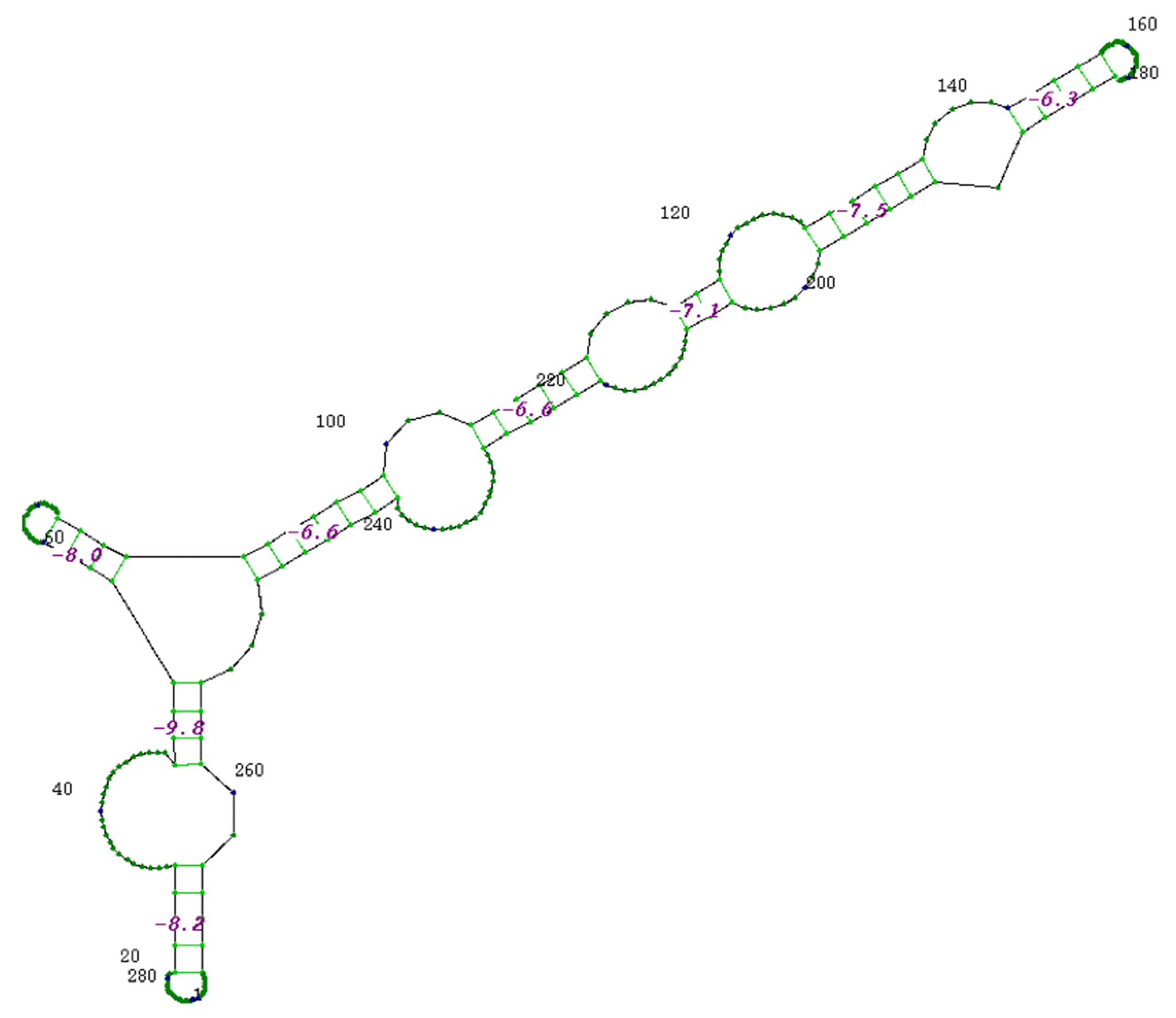

Supplement: Figure S12 — Predicted secondary structure of hsrω ncRNA 280 bp repeat unit. Secondary structure prediction of the 280b hsrω-n ncRNA repeat unit used for the binding and ATPase assays (Figure 5). Secondary structure prediction was obtained using the RNA secondary structure prediction tool present at the GeneBee molecular biology server (http://www.genebee.msu.su/services/rna2_reduced.html). (TIF) [file pgen.1002096.s012.tif]

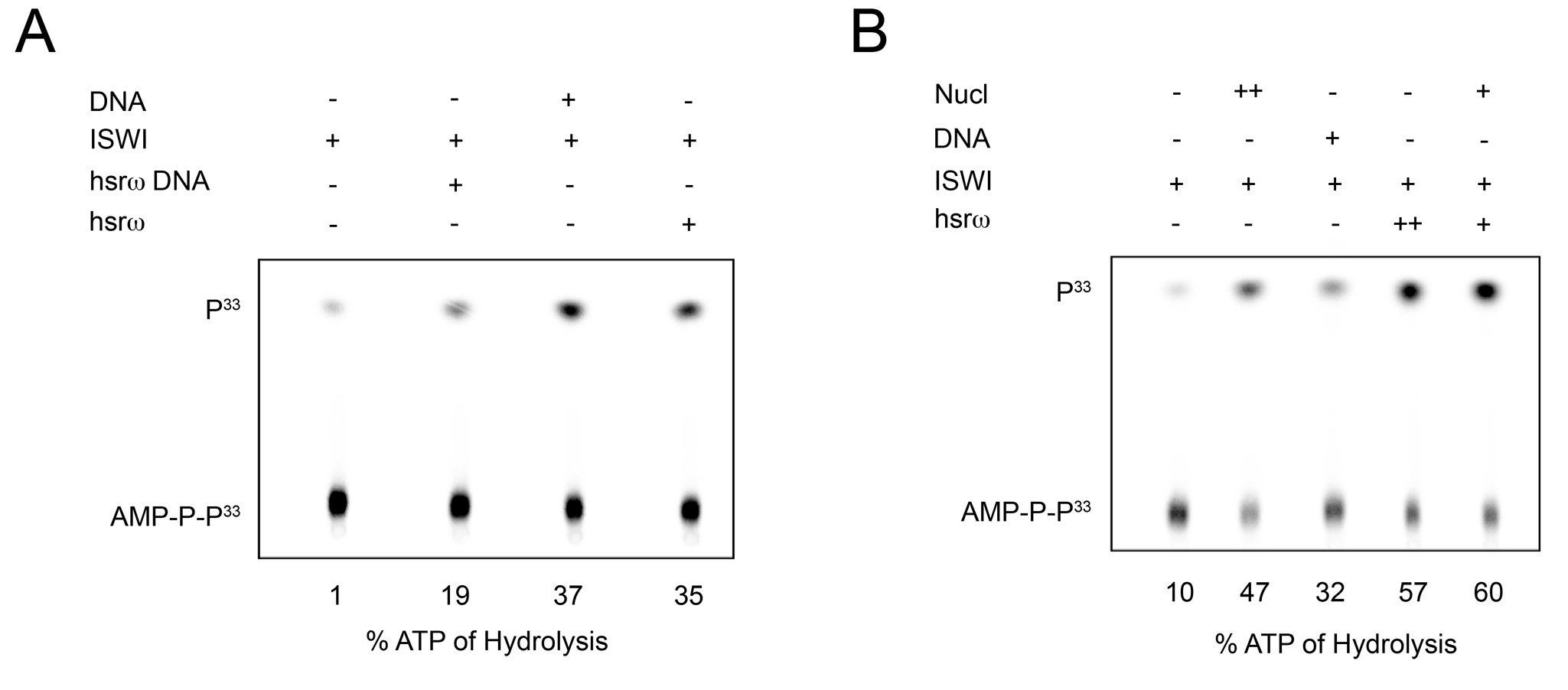

Supplement: Figure S13 — Effect of hsrω encoding DNA and of nucleosomes on hsrω-stimulated ISWI ATPase activity. (A) Stimulation of the ATPase activity of ISWI by generic plasmid DNA, linear double stranded 280 bp DNA encoding the hsrω-n repeat unit or in vitro transcribed 280b repeat unit RNA was assayed in vitro by thin layer chromatography in the presence of radioactive ATP-γ33P. Samples were incubated for 30 minutes. (B) Effect of nucleosome presence on the hsrω-stimulated ISWI ATPase activity. Samples were incubated for 60 minutes. When nucleosomes (Nucl) and hsrω ncRNA were combined in the same reaction, the amount of each was half of the amount used to test them alone. Stimulation of the ATPase activity by the used nucleic acids assayed is noted below each lane as percentage of the ATP hydrolysed (P33 = P33 radioactive labeled hydrolyzed gamma phosphate, AMP-P-P33 = radioactive labeled non-hydrolyzed ATP). (TIF) [file pgen.1002096.s013.tif]
