# Supplementary material for: The ISWI Chromatin Remodeler Organizes the hsrω ncRNA–Containing Omega Speckle Nuclear Compartments
Source: PLoS Genet. 2011 May 26;7(5):e1002096. doi: 10.1371/journal.pgen.1002096 (PMC3102753; doi:10.1371/journal.pgen.1002096)
Supplement: Table S1 — (A) Results of the genetic interaction test between hsrω and sqd alleles with ISWI, as revealed by the ISWIEGUF eye test (see also Figure S1 and Text S1). The nature of the alleles tested has been obtained from the Flybase (www.flybase.org); a – sign in column 2 indicates that nature of the allele is not know. (B) RNAi based reduction of hsrω transcripts using either the ey-GAL4 or the Act5C-GAL4 driver prolongs the survival of ISWI trans-heterozygous null mutants to pupal stage (also see Figure 1K), though none of these pupae enclose as flies. n = number of larvae scored for each genotype. (DOC) [file pgen.1002096.s014.doc]

Onorati et al Table S1

**A**

| **Allele Tested** | **Allele Nature** | ***ISWI ^EGUF^* Phenotype** |
| --- | --- | --- |
|  |  |  |
| *hsrω^EP3115^* | loss | ***Suppressed*** |
|  |  |  |
| *hsrω ^RNAi3^* | loss | ***Suppressed*** |
|  |  |  |
| *hsrω^DG16301^* | - | ***Suppressed*** |
|  |  |  |
| *hsrω^e01850^* | - | ***Suppressed*** |
|  |  |  |
| *hsrω^EP3037^* | - | ***Suppressed*** |
|  |  |  |
| *hsrω^EP93D^* | gain | ***Enhanced*** |
|  |  |  |
| *Sqd ^EP3631^* | loss | ***Suppressed*** |
|  |  |  |
| *Sqd ^e01416^* | - | ***Suppressed*** |
|  |  |  |
| *Sqd ^c04803^* | - | ***Suppressed*** |
|  |  |  |
| *Sqd ^f01931^* | - | ***Suppressed*** |
|  |  |  |

B

| **Genotype** | **n** | **Pupae** |
| --- | --- | --- |
| *ISWI^1^/ISWI^2^; eyGal4/UAS-hsrω^RNAi^* | 54 | 40 |
| *ISWI^1^/ISWI^2^; eyGal4/+* | 50 | 2 |
| *ISWI^1^/ISWI^2^; UAS-hsrω^RNAi^/+* | 53 | 0 |
| *ISWI^1^/ISWI^2^; ACT5CGal4/UAS-hsrω^RNAi^* | 51 | 26 |
| *ISWI^1^/ISWI^2^; ACT5CGal4/+* | 51 | 1 |
| *ISWI^1^/ISWI^2^; UAS-hsrω^RNAi^/+* | 52 | 0 |
