# Supplementary material for: The ISWI Chromatin Remodeler Organizes the hsrω ncRNA–Containing Omega Speckle Nuclear Compartments
Source: PLoS Genet. 2011 May 26;7(5):e1002096. doi: 10.1371/journal.pgen.1002096 (PMC3102753; doi:10.1371/journal.pgen.1002096)
Supplement: Text S1 — Supporting Materials and Methods. (DOC) [file pgen.1002096.s015.doc]

**Supporting Materials and Methods**

**FRISH and Immuno-FRISH on whole nuclei**

The desired tissues (salivary glands, Malpighian tubules, testes) from late third instar larvae of the desired genotype were dissected, under RNAse-free conditions in PBS and immediately fixed in freshly prepared 3.7% paraformaldehyde (in PBS) for 3min at 24^o^C (RT). After 3 washes (2 min each) in PBS, the tissue was transferred in a drop of PBS on a clean RNAse-free slide and after replacing PBS with a small drop of 50% acetic acid, a coverslip was put over the tissue. Squashing was carried out by placing slide with the coverslip between folds of clean and fibre-free filter paper onto which a bottle with 1 liter water was placed for ten seconds. No other manual pressure was applied. Slides with partially squashed tissue were immediately flash frozen in liquid nitrogen and the coverslips were flipped off with a sharp blade. Slides were immediately put in 50% ethanol for 5 min and dehydrated through 70%, 90% and 100% ethanol series and finally air dried at RT. Dried slides were treated with 0.1% DEPC in PBS for 3 min, washed in PBS and re-fixed in freshly prepared 3.7% paraformaldehyde (in PBS) for 3min at RT (the 2^nd^ fixation helps better preservation of the nuclear architecture). Finally, the slides were washed in PBS 3 times (2 min each), dehydrated through 70%, 90% and 100% ethanol series and air dried. They were used immediately for fluorescence RNA:RNA in situ hybridization (FRISH) and/or immunostaining. For Immuno-FRISH, they were first processed for immunostaining as previously described [11], taking precaution for RNAse-free working environment. All the solutions were prepared with DEPC treated water and the blocking solution (PBS1X, NaN_3_ 0,02%, Triton X-100 0,1%) was without bovine serum albumin (BSA). Slides were mounted with DAPI-containing mounting medium (Vectashield) and were analyzed under a LEICA DM4000 ﬂuorescence microscope or an Olympus IX70 confocal microscope.

**Heat shock**

Third instar larvae were transferred to preheated 1.5-ml microcentrifuge tube. The tubes were submerged in a 37°C water bath for 40 min. After the heat shock, tubes were kept at RT for 20 minutes to allow the larvae to recover before dissection.

**RNA-Immunoprecipitation**

Native larval nuclear protein extracts from third-instar *w^1118^* larvae were prepared as previously described [11]. Larval Nuclear Buffer I and Larval Nuclear Buffer II were both supplemented with 100 μg/ml of RNAse inhibitor (Applied Biosystem). ISWI immunoprecipitation was performed on the nuclear protein extracts using protein A/G plus-agarose beads (Sigma) and affinity purified ISWI antibody [4]. 25 µl of beads were resuspended in 75 µl IB buffer (10 mM Hepes-KOH, pH 8.0, 1 mM EDTA, 10% Glycerol, 50 mM NaCl) and incubated with 2 µg of anti-ISWI or generic rabbit IgG (Santa Cruz Biotechnology), as negative control, for 1 hour at RT with gentle agitation. The beads were centrifuged for 2 minutes at 4000g and washed three times each with 500 µl of IB buffer and finally incubated with 250 μg of wild-type nuclear protein extracts in 125 µl final volume of IP buffer (10 mM Hepes-KOH, pH 8.0, 100 mM NaCl, 10% Glycerol, 0.,05% TWEEN 20, 100 µg/ml PMSF, complete protease inhibitors-Roche 1X) over-night at 4°C with gentle agitation. The beads were pelleted as before and washed five times with 1.2 ml of IP buffer for 10 minutes each under rotation at room temperature. Finally, the bound material was split in two and eluted either with 15 µl SDS-PAGE sample buffer for Western blotting analysis or with 100 µl of TEL buffer (200 mM Tris-HCl, pH 7.4, 25 mM EDTA, pH 8.0, 100 mM LiCl, 1% SDS) for RT-PCR.

For Western blotting, proteins were separated by SDS-PAGE, blotted and challenged with the ISWI antibody. Primary antibody binding was detected using horseradish-peroxidase-coupled secondary antibody (Bio-Rad) and Super Signal chemiluminescent reagent (Pierce). For RT-PCR, the RNA was extracted from 25 µl each of the TEL buffer added beads, the unbound or the immunoprecipitated material with 600 µl of TRIzol (Invitrogen); the RNA was precipitated overnight with 0.7 volumes of isopropanol at -20°C. The pellet was washed in 75% cold ethanol, dissolved in 10 µl RNAse free water and incubated for 15 minutes at 37°C. The first-strand cDNA was synthesized using MuLV reverse transcriptase (Applied Biosystem) and 1µl of random hexamers (Applied Biosystem). 4 µl of the reaction mixture was used for PCR amplification using the hsrω-n RNA specific primer pairs that amplify the 280bp unit of tandem repeats in the hsrω-n transcript (hsrω forward, 5’-CGAAAAGGCTTATCCTCTTGGTAAA-3’, and hsrω reverse, 5’-AAGGATAATGATTAAGGTAATCGGG-3’), the Act5C transcript (ACT5C forward, 5’-CACGGTATCGTGACCAACTG-3’, Act5C reverse, 5’-GCCATCTCCTGCTCAAAGTC-3’), the U4 ncRNA (U4 forward, 5’-GCAGAGGCGATATCGTAACC -3’, U4 reverse, 5’- GCTTCCAAAAATTGCCGTAG -3’) or the Rox1 ncRNA (Rox1 forward, 5’- CCCAGAAGAAACTGCCACTGC -3’, Rox1 reverse, 5’- AATGTCCCTTTTCGAGCG -3’). For PCR amplification of hsrω and Act5C transcripts we used the following programme: 94°C 2min, 30 cycles (94°C for 30sec, 50°C for 30sec, 72°C for 30sec) and a final extension at 72°C for 2min. To amplify the U4 transcript we instead used the following programme: 94°C 4min, 30 cycles (at 94°C for 30sec, at 46°C for 30sec, at 72°C for 1 min) and a final extension at 72°C for 10min. Finally for the PCR amplification of Rox1 transcript, we used: 94°C 4min, 30 cycles (at 94°C for 30sec, at 55°C for 30sec, at 72°C for 1 min) and a final extension at 72°C for 10min. The PCR products were analyzed by agarose electrophoresis and the ethidium bromide stained gel images were acquired with the ChemiDoc XRS imager (BioRad).

**CLIP (Cross-Linking & Immuno Precipitation)**

Fixed larval nuclear extracts were prepared according to a previously published protocol for the production of native larval nuclear extract [35], with some key modifications. Larval Nuclear Buffer I (LNB1RI) was supplemented with 100 units/ml of RNAse inhibitor (Applied Biosystems) and all precautions were taken for RNAse-free conditions (i.e. use of DEPC treated water to make all solutions). Batches of 0.5 grams of third-instar wild type (*w^1118^*) larvae were collected and washed with Larval Wash Buffer (LWB: 0.7%NaCl, 0.1%TritonX-100). Dry larvae were resuspended in 2 ml of LNB1RI per gram of larvae. Larvae were homogenized as previously described [35]. The homogenate was filtered through a layer of 64 µm Nitex nylon mesh (Genesee Scientific). The cells and nuclei were transferred to a pre-chilled glass Dounce homogenizer (Wheaton) and 8 full strokes were applied with a tight “B” pestle in order to break up the remaining larval cells. Free nuclei were then centrifuged at 4000 g for 5 min at 4°C. The lipid layer on the top of the tube was carefully removed with a small metal spatula. The supernatant was removed and the nuclear pellet was washed three times under rotation for 3min each in 1X PBS_DEPC_ followed by centrifugation at 1100g for 2 min at 4°C. After the last wash, nuclei were resuspended in 1X PBS_DEPC_- with 1% Formaldehyde and incubated for 15 min under rotation. Crosslinking reaction was quenched by the addition of glycine (pH 7.0) to a ﬁnal concentration of 0.25M followed by incubation at room temperature for 5 min. The nuclear pellet was collected by centrifugation at 1100g for 2min at 4°C, washed twice with cold 1X PBS_DEPC_. The nuclear pellet was resuspended in 5ml Nuclear Extraction Buffer (15mM Hepes pH=7.0, 5mM MgCl2, 0,2 mM EDTA, 0,5 mM EGTA, 10mM KCl, 350mM Sucrose (added fresh), 0,1%Tween-20, 1mM DTT (added fresh)) and vortexed for 10 sec. Nuclear Extraction Buffer was added to make 10ml volume. The sample was centrifugated at 3220 g for 5 min at 4°C, the supernatant was removed and the crosslinked pellet was resuspended in 2ml of RIPA buffer (50mM Tris-HCl pH 7,5, 1% NP40, 0,5% Na-deoxycholate, 0,05% SDS, 1mM EDTA, 150mM NaCl) containing protease inhibitors (complete, mini, EDTA-free protease inhibitor cocktail tablet, Roche). The resuspended pellet was sonicated three times for 20 sec each with the sample being kept on ice for 2 min between each step of sonication. The insoluble material was removed after centrifugation at 16000g for 10 min at 4°C. CLIP was conducted as previously described [1], with 250μl of the supernatant, containing the fixed larval nuclear extract and 1μg of the affinity purified anti-ISWI antibody [4].

**RNA Pull Down**

The RNA pull down assay was performed as previously published [2] using 3μg of *in vitro* transcribed hsrω-n 280b repeat unit [32] and 1mg of larval nuclear extract. Native larval nuclear protein extracts from third-instar *w^1118^* larvae were prepared as previously described [11]. Larval Nuclear Buffer I and Larval Nuclear Buffer II were both supplemented with 100 μg/ml of RNAse inhibitor (Applied Biosystem).

**RT-PCR using tissue RNA**

Total RNA was puriﬁed from salivary glands and Malpighian tubules of third instar larvae of the desired genotype, using TRIzol reagent following the manufacturer’s (Invitrogen) recommended protocol. First strand cDNA was synthesized using MuLV reverse transcriptase (Applied Biosystems); 4 µl of the reaction mixture was used as template for PCR ampliﬁcation, using two different primer combinations for the hsrω-n RNA. In one set of reactions, the above noted primer pairs for the 280b unit of tandem repeats of hsrω-n RNA were used with the thermal cycle parameters described above. The second pair of hsrω primers were designed to discriminate between the unspliced and spliced forms of the hsrω-n transcripts (forward, 5’ ggcagacatacgtacacgtggcagcat, and reverse, 5’ACCAAGAGGCTAATCGAC) with the PCR cycle parameters being, 4 min at 94°C followed by 35 cycles of 45 sec at 94°C, 45 sec at 60°C, 45 sec at 72°C and final extension for 2 min at 72°C. The PCR products were analyzed by agarose electrophoresis and the images were acquired with the ChemiDoc XRS imager (BioRad). The ISWI RNA was amplified along with the above two hsrω specific primer pairs, using the same thermal cycle parameters; the ISWI mRNA-specific primers were: forward, 5’AAGCAGCCTATCGTTCAGGA and reverse, 5’CCCGACCGTACTTTTCGTTA. The Act5C RNA was amplified as loading control with the earlier noted primer pairs and thermal cycle parameters.

**Northern Blotting and hybridization**

About 15μg each of total RNA samples were separated on a formaldehyde/formamide denaturing 1% agarose gel for 5 hours at 30mA in MOPS. The RNA was transferred to a nitrocellulose membrane (Whatman-Protran) by overnight capillary transfer at room temperature using RNAse-free 20XSSC (Sodium Citrate, Dihydrate 8,8%, Sodium Chloride 17,5%) as the transfer buffer. The transferred RNA was crosslinked using the UVC 500 crosslinker (Amersham) and the membrane was prehybridized for 3 hours in HybBuffer (10 ml solution contained 5ml Formamide, 2.5ml 20XSSC, 1ml 50X Denhardt’s 10%, 0,5 ml 10% SDS and 200 μl ssDNA 10mg/ml) at 42°C with rotation. A random primed (DNA labeling kit; Roche) radiolabeled DNA probe (dCTPα^32^-P) corresponding to the 280 bp unit of tandem repeats of the hsrω-n transcript was prepared with α^33^P-dATP; the unincorporated nucleotides were removed with Mini Quick Spin™ DNA Column (Roche). The membrane was incubated overnight with fresh HybBuffer with the radiolabeld probe at 42°C with rotation. Following hybridization, the membrane was washed twice for 10min each in 2XSSC (at 42°C) and once in 1XSSC at 42°C for 10 min. After air drying the membrane, the hybridization signal was recorded using the Personal Molecular Imager System (BioRad).

**Fluorescence in situ hybridization (FISH) with oligodT-Cy3**

FISH with oligodT-Cy3 was performed as previously published [3] with minor modifications. Specifically, larval salivary glands were dissected in physiological solution (0.7% NaCl) and fixed with 3.7% paraformaldehyde in PBS for 10 min. After fixation, salivary glands were washed in PBS for 5min and permeabilized with PBS containing 0.5% Triton X-100 (10 min) at RT and washed again in PBS for 5min. For detection of the poly(A)+ RNAs, the glands were incubated for 30 min at 37°C in prehybridization buffer (2XSSC, 20% formamide, 0.2% BSA, 1 mg/ml yeast tRNA). For hybridization, the glands were transferred to a humidified chamber and incubated in 20 μl of hybridization buffer (prehybridization buffer + 10% dextran sulfate) containing 0.5 pmol/ml oligo(dT)50 fluorescently end-labeled with Cy3 molecules (Gene Link). The glands were hybridized for 3h at 37°C and washed twice, 5min each in 2XSSC, 20% formamide at 42°C, once for 5min in 2XSSC at 42°C, once for 5 min in 1XSSC, and finally once for 5 min in PBS. Finally, the glands were mounted with DAPI-containing mounting medium (Vectashield) and analyzed by fluorescent microscopy (LEICA DM4000).

**Supporting References**

1. Niranjanakumari S, Lasda E, Brazas R, Garcia-Blanco MA (2002) Reversible cross-linking combined with immunoprecipitation to study RNA-protein interactions in vivo. Methods 26: 182-190.

2. Tsai MC, Manor O, Wan Y, Mosammaparast N, Wang JK, et al. Long noncoding RNA as modular scaffold of histone modification complexes. Science 329: 689-693.

3. Piacentini L, Fanti L, Negri R, Del Vescovo V, Fatica A, et al. (2009) Heterochromatin protein 1 (HP1a) positively regulates euchromatic gene expression through RNA transcript association and interaction with hnRNPs in Drosophila. PLoS Genet 5: e1000670.
